# Rethinking Osteoarthritis Management: Synergistic Effects of Chronoexercise, Circadian Rhythm, and Chondroprotective Agents

**DOI:** 10.3390/biomedicines13030598

**Published:** 2025-02-27

**Authors:** Eloy del Río

**Affiliations:** Independent Researcher, 11520 Cádiz, Spain; eloy.delrio@uca.es

**Keywords:** osteoarthritis, cartilage proteoglycan degradation, aggrecanases, disease-modifying osteoarthritis drugs (DMOADs), pharmacokinetics, circadian rhythms/chronobiology, chronoexercise, chronotherapy/chronomedicine

## Abstract

Osteoarthritis (OA) is a chronic and debilitating joint disease characterized by progressive cartilage degeneration for which no definitive cure exists. Conventional management approaches often rely on fragmented and poorly coordinated pharmacological and non-pharmacological interventions that are inconsistently applied throughout the disease course. Persistent controversies regarding the clinical efficacy of chondroprotective agents, frequently highlighted by pharmacovigilance agencies, underscore the need for a structured evidence-based approach. Emerging evidence suggests that synchronizing pharmacotherapy and exercise regimens with circadian biology may optimize therapeutic outcomes by addressing early pathological processes, including low-grade inflammation, oxidative stress, and matrix degradation. Recognizing the influence of the chondrocyte clock on these processes, this study proposes a ‘prototype’ for a novel framework that leverages the circadian rhythm-aligned administration of traditional chondroprotective agents along with tailored, accessible exercise protocols to mitigate cartilage breakdown and support joint function. In addition, this model-based framework emphasizes the interdependence between cartilage chronobiology and time-of-day-dependent responses to exercise, where strategically timed joint activity enhances nutrient and waste exchange, mitigates mitochondrial dysfunction, supports cellular metabolism, and promotes tissue maintenance, whereas nighttime rest promotes cartilage rehydration and repair. This time-sensitive, comprehensive approach aims to slow OA progression, reduce structural damage, and delay invasive procedures, particularly in weight-bearing joints such as the knee and hip. However, significant challenges remain, including inter-individual variability in circadian rhythms, a lack of reliable biomarkers for pharmacotherapeutic monitoring, and limited clinical evidence supporting chronoexercise protocols. Future large-scale, longitudinal trials are critical to evaluate the efficacy and scalability of this rational integrative strategy, paving the way for a new era in OA management.

## 1. Introduction

Osteoarthritis (OA) is the most prevalent joint disorder worldwide, affecting over 595 million individuals and imposing a considerable economic and societal burden [1]. Direct OA-related healthcare costs, including joint replacement surgeries and long-term care, contribute to billions of dollars annually. Beyond its financial impact, OA significantly impacts quality of life, as individuals often experience chronic pain, functional impairment, and limitations in both daily and social interactions. Furthermore, OA is commonly associated with other conditions such as stroke, hypertension, gastrointestinal disorders, anxiety, and depression [2]. Although increased OA severity is linked to the presence of multiple comorbidities, it remains uncertain whether these conditions precede OA, result from it, or interact in a bidirectional manner [3]. Despite the widespread and severe consequences of OA, its incidence continues to rise at an alarming rate, primarily driven by aging populations in developed nations, particularly those with large baby boom cohorts. Recent findings from the Lancet Commission emphasize the escalating nature of this global crisis [1], with the burden of OA increasing significantly rather than stabilizing. According to Wallace et al. [4], the prevalence of knee OA has doubled since the mid-20th century, a trend expected to persist in the coming decades. Recent projections indicate an exponential increase in the demand for total joint replacements between 2040 and 2060 [5], further exacerbating the strain on healthcare resources. This dramatic rise in OA-related healthcare demands presents a critical challenge as healthcare systems may struggle to maintain long-term sustainability in the face of escalating costs. Without a shift toward more proactive and sustainable management strategies, the growing prevalence of OA and its associated healthcare costs are likely to become increasingly unsustainable, leading to further strain on healthcare resources and abab diminished quality of life for the affected individuals. The increasing incidence of early onset OA among younger populations exacerbates these challenges, introducing additional complexities in both diagnosis and management. Current treatment strategies, including pharmacological approaches such as analgesics, nonsteroidal anti-inflammatory drugs (NSAIDs), corticosteroids, and opioids, are largely aimed at managing symptoms, offering limited solutions for halting disease progression, or addressing the underlying pathophysiological mechanisms. Despite significant advancements in our understanding of the OA pathophysiology, effective disease-modifying therapies remain elusive, leaving patients trapped in a cycle of chronic pain and disability [6]. To address this growing public health crisis, a paradigm shift toward earlier interventions and preventive care is essential. Implementing these strategies may not only reduce the long-term burden of OA on healthcare systems, but also significantly improve patient outcomes and quality of life in future generations.

Cutting-edge research to understand the initial triggers and early molecular changes in OA is essential develop targeted interventions aimed at preventing severe symptoms and reducing the need for surgical treatment [7,8,9,10]. Traditionally considered a simple wear-and-tear disease, OA is now recognized as a complex condition influenced by non-modifiable factors such as age, genetics, and sex, as well as modifiable factors such as obesity, joint injuries, and physical inactivity [11,12,13]. How can we explain why our expanding knowledge has not yet led to a decline in OA incidence? This paradox—in which the accumulation of knowledge and research has not yet translated into a decrease in incidence—demands radical rethinking, and highlights the critical need for a more effective integration of this understanding into clinical practice and public health strategies. To address this challenge, the current study offers a fresh perspective—derived from an extensive review of the literature on OA management by combining physical activity/exercise, circadian rhythm regulation, and the strategic use of orally administered chondroprotective agents. By examining the interplay among these factors, I aimed to develop a comprehensive strategy that addresses the multifactorial nature of cartilage care. Although conventional chondroprotective agents, such as glucosamine and chondroitin sulfate, have shown promise in supporting cartilage integrity and slowing OA progression, their clinical efficacy remains inconsistent, raising concerns regarding their isolated application [6,7,9,14,15,16]. Despite their widespread use and encouraging preclinical findings, the variable translation of these agents in delivering consistent clinical outcomes in human studies continues to drive debates. This underscores the need for multifaceted approaches that integrate multiple therapeutic dimensions, including lifestyle modifications, physical activity levels (PALs), and exercise science principles [17]. Emerging research in circadian biology (or chronobiology) also suggests that synchronizing interventions with biological rhythms can significantly enhance the effectiveness of these pharmacological therapies [18,19,20]. Specifically, chronoexercise, or the alignment of physical stimuli with circadian rhythms, may enhance the protective effects of exercise and medical treatments. Similarly, chronotherapeutics—the strategic timing of drug administration to maximize therapeutic effects—remains underexplored in the context of OA. Recent medical hypothesis research highlights that carefully optimizing the timing of interventions has the potential to significantly improve therapeutic outcomes, providing a promising avenue for future clinical advancements [17]. However, this field is still far from mature, and translating these findings into clinical practice remains in its infancy, even though it holds significant promise for future advancement.

Moving forward, this perspective introduces an integrative framework for OA management, designed to promote cartilage preservation, slow disease progression, and decrease reliance on invasive treatments, particularly when applied in the early stages of OA. By aligning therapeutic interventions with biological rhythms, this circadian-based precision medicine approach aims to improve patient outcomes and reduce the long-term healthcare and societal burden of OA. However, its practical implementation and clinical value will require rigorous validation through randomized longitudinal large-scale clinical trials to evaluate its efficacy and safety.

## 2. Preserving Cartilage Integrity: Critical Insights into Composition, Structure, and Function in Joint Health and Disease

Hyaline cartilage is a highly specialized connective tissue that covers the ends of bones in synovial joints, enabling smooth, low-friction movement and the effective distribution of mechanical loads [21,22,23]. Its unique anatomical composition is optimized to withstand the significant pressures typical of weight-bearing joints, such as the knees and hips, thereby ensuring durability and resistance to wear [24,25]. This articular cartilage primarily consists of chondrocytes, the extracellular matrix (ECM), and water [22]. The ECM is predominantly composed of type II collagen and proteoglycans, which are essential for maintaining mechanical support. Collagen fibers provide tensile strength and establish a supportive framework for the ECM [26], whereas proteoglycans, particularly aggrecan, bind to hyaluronic acid to form large aggregates that enhance compressive resilience in the tissue [27,28]. Aggrecan, a key structural proteoglycan composed of globular domains (G1, G2, and G3) and polysulfated glycosaminoglycan-rich regions, is vital for the ability of cartilage to withstand compressive forces because of its water retention capacity, which improves its elasticity and shock absorption [28,29]. This distinctive chemical and structural composition not only provides tensile strength and compressive resistance but also contributes to the gel-like consistency of the cartilage, enabling it to retain significant amounts of water, approximately 65–80% of its wet weight. A high water content is critical for preserving biomechanical properties, enabling effective shock absorption, and supporting nutrient diffusion within the tissue in the absence of blood vessels [30]. Additionally, non-collagenous proteins, including small leucine-rich proteoglycans (SLRPs) such as decorin, biglycan, and fibromodulin, along with multiadhesive matrix glycoproteins, such as cartilage oligomeric matrix protein (COMP) and fibronectin (FN), are essential components of the ECM and contribute to its structural integrity and functional maintenance. SLRPs regulate collagen fibrillogenesis, contributing to ECM stability and tissue resilience, whereas multiadhesive glycoproteins facilitate key interactions between cells and the ECM, supporting matrix assembly as well as cellular adhesion, migration, and signaling [27,31,32,33,34,35,36,37,38,39]. Together, these proteins not only support ECM organization but also play a pivotal role in maintaining tissue architecture, regulating cellular communication, and coordinating tissue repair and homeostasis. These fundamental features of the cartilage structure and composition are crucial for preserving joint integrity and preventing degenerative changes associated with OA.

**Figure 1:**
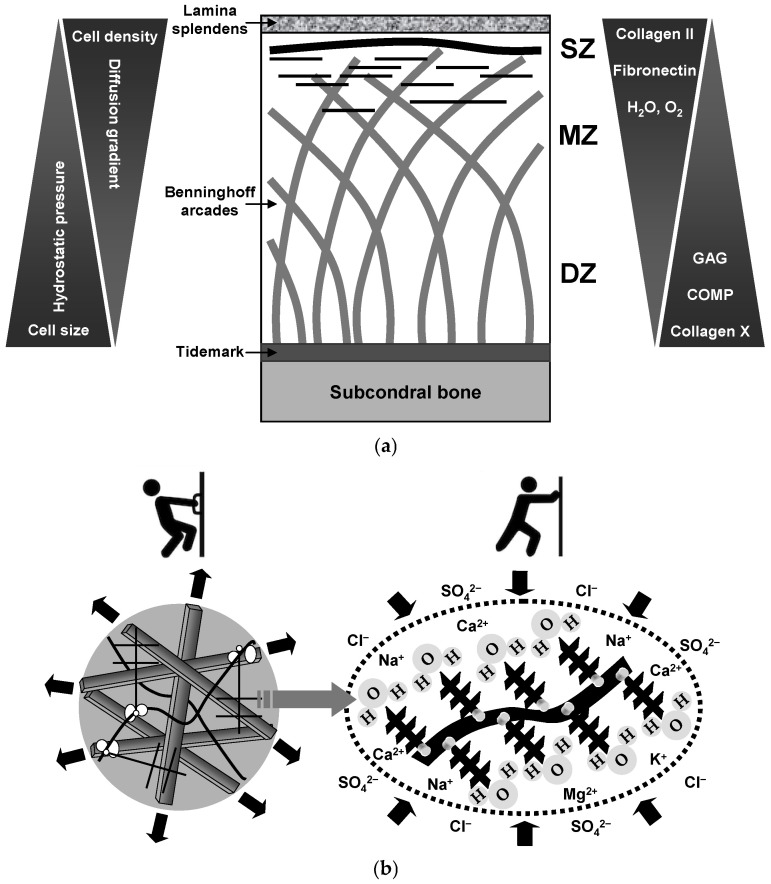
(**a**) Schematic representation of the distinct zonal organization within the mature hyaline cartilage of adult diarthrodial joints, depicting the structural features and specialized functions of the superficial (tangential), middle (transitional), and deep (radial) zones. This illustration highlights the collagen fiber orientation across these zones, which together contribute to the mechanical properties and joint integrity of the cartilage. In the superficial zone, densely packed collagen fibers are aligned parallel to the articular surface, providing essential tensile strength and resistance to shear forces. The middle zone marks a transitional layer where the organization of collagen fibers becomes more random, aiding the distribution of loads and resilience against compressive forces. In the deep zone, collagen fibers are oriented perpendicularly, anchoring the cartilage to the underlying subchondral bone and enhancing compressive stiffness. A gradual increase in proteoglycan concentration and a decrease in water content with depth further support mechanical resilience and influence nutrient diffusion. Additionally, the figure shows a decrease in chondrocyte density from the superficial to the deep zones, emphasizing the distinct cellular functions involved in maintaining cartilage integrity and adaptation to mechanical loading. (**b**) Biomechanical equilibrium in joint cartilage: Interdependent roles of proteoglycans and collagen fibers (shown diagrammatically). This figure illustrates the dual-mechanism system within cartilage, emphasizing the interconnected mechanisms of collagen tension (**left**) and osmotic swelling pressure from the proteoglycan aggregate (**right**), which together contribute to the tensegrity of cartilage and preserve its mechanical stability. The collagen fiber network, depicted on the left, provides tensile strength that resists deformation and maintains structural integrity. Conversely, the proteoglycan matrix, primarily composed of aggrecan, absorbs water and generates osmotic swelling pressure, which alleviates compressive forces and enhances cushioning. In this context, salt and water work synergistically, with aggrecan serving as the principal load-bearing component through mechanisms of charge repulsion and Donnan equilibrium, facilitating solute influx and swelling pressure generation. The collagen II network limits swelling and ensures its stability. This complex dynamic interaction between the tensile and compressive forces is essential for the resilience of cartilage, enabling effective shock absorption and supporting optimal joint functionality. Abbreviations: SZ, superficial zone; MZ, middle zone; DZ, deep zone; GAG, glycosaminoglycan; COMP, cartilage oligomeric matrix protein.

As illustrated in Figure 1a, the structural organization of hyaline cartilage provides critical insight into its resilience and susceptibility to degeneration. Organized into four distinct zones—superficial (tangential), middle (transitional), deep (radial), and calcified—hyaline cartilage exhibits unique cellular and ECM compositions and structural arrangements within each zone, which support their specialized functions [21,22,23,26,40,41,42,43]. The superficial zone, which contains abundant collagen fibers aligned parallel to the articular surface, is designed to resist shear forces and protect the underlying layers [26]. The middle zone, characterized by randomly oriented collagen fibers, acts as a transitional layer between the superficial and deep zones, contributing to the distribution and absorption of compressive loads. In the deep zone, collagen fibers oriented perpendicular to the surface anchor the cartilage to the subchondral bone, providing significant resistance to compressive forces. The calcified zone, located beneath the deep zone, integrates the cartilage with the subchondral bone, facilitating mechanical continuity between these softer and harder tissues. Maintaining the integrity of vertical collagen fibers in the deep zone is essential for preserving cartilage durability [41]. Disruption of this fibrillar network, particularly near the tidemark [44,45,46], compromises load distribution and increases the risk of mechanical failure. This complex architectural framework not only supports the mechanical resilience of cartilage, but also underscores its susceptibility to degenerative processes, making it a key focus in efforts to maintain joint health over time.

The mechanical behavior of hyaline cartilage is critically Influenced by its anisotropic, orthotropic, and inhomogeneous structural characteristics, which vary with depth and anatomical location within joint tissue [21,47]. Under compressive loading, the negatively charged glycosaminoglycan chains within proteoglycans create osmotic swelling pressure, which contributes significantly to tissue stiffness. This swelling pressure is counterbalanced by the tensile strength of the collagen network, particularly through the “arcade-like” organization of collagen fibers, first described by Benninghoff [48]. In this configuration, collagen fibers form a distinctive arched pattern, especially in the deep zone, where they align perpendicularly to the articular surface and curve downward into the calcified cartilage (Figure 1a). This orientation enables the fibers to anchor securely into the subchondral bone, enhancing the structural integrity of the cartilage–bone interface. The arcade-like fiber structure plays a critical role in distributing compressive forces and minimizing shear stress, which are essential for protecting the tissue from mechanical failure. Additionally, the tensile strength provided by this collagen architecture plays a crucial role in maintaining cartilage integrity, particularly under repetitive mechanical stresses experienced during normal weight-bearing activities [21,23,26,41]. This precise balance between the osmotic swelling pressure from proteoglycans and the tensile support of the collagen network allows cartilage to deform and rebound as required, supporting its function as a durable, load-distributing tissue within the joint. These biomechanical properties and structural adaptations are essential for ensuring the long-term resilience of cartilage and preserving joint health under physiological conditions.

The biomechanical stability of hyaline cartilage, as schematically depicted in Figure 1b, relies on a highly coordinated interaction between collagen fibers and the proteoglycan-enriched ECM [49,50]. Collagen fibers provide tensile strength, resist deformation, and maintain the structural integrity under mechanical loading. In parallel, the proteoglycan matrix, primarily composed of aggrecan and other polysulfated polysaccharides, attracts water, generating osmotic swelling pressure that counteracts compressive forces. This swelling pressure arises from the fixed charge density of the ECM [49,50], a property generated by densely packed negatively charged glycosaminoglycans in aggrecan molecules. The fixed charge density establishes a Donnan equilibrium, promoting water and solute influx, whereas charge repulsion mechanisms within the ECM enhance the resistance of the tissue to compression. This intricate balance between the compressive and tensile forces is critical for cartilage resilience, enabling effective shock absorption, uniform load distribution, and smooth joint articulation. The specialized biochemical and structural properties of articular cartilage support its ability to deform under loads, allowing it to release water and alter its shape to distribute pressure evenly across the synovial joint [51,52]. Upon load release, cartilage reabsorbs water, regaining its original shape, a process that is reflective of its viscoelastic properties. These diurnal fluctuations in water content—where cartilage expels water during daily compressive activities and reabsorbs it during nocturnal rest—are essential for maintaining hydration, supporting nutrient and waste exchange, and preserving biomechanical properties. This dynamic water exchange process enables cartilage to remain resilient under varying physiological demands. Collectively, these structural and electrochemical properties are critical for safeguarding the integrity and functionality of cartilage.

In addition to its macromolecular structure, the biomechanical stability of cartilage is intricately regulated by a delicate balance of inorganic ion concentrations within the ECM, particularly sodium (Na^+^), calcium (Ca^2+^), and potassium (K^+^) [53,54,55,56,57,58,59,60,61]. These ions are fundamental for molecular interactions that support cartilage function, and even small variations in their levels can lead to significant effects on cartilage stability and performance. For instance, increased Ca^2+^ levels have recently been implicated in the early stages of cartilage degradation [59] and are known to disrupt the structural, mechanical, and frictional properties of the lamina splendens, an acellular ultra-thin superficial cartilage layer (Figure 1a), leading to matrix breakdown and OA onset. Similarly, maintenance of a low intracellular Na^+^/K^+^ ratio, achieved primarily through the action of Na^+^/K^+^-ATPase, is crucial for chondrocyte function, with alterations in the extracellular Na^+^ concentration influencing the expression and plasma membrane density of this pump, thereby modulating the ionic balance essential for cartilage homeostasis [57]. Moreover, fluctuations in the free Na^+^ concentration, driven by mechanical compression and the redistribution of water across regions with varying levels of stiffness, modulate ionic interactions within the pericellular matrix, often referred to as the chondron, a structurally distinctive microdomain crucial for maintaining cell homeostasis and mediating the response to mechanical stimuli [53,56,58,60,61,62]. These changes can modify the binding affinity of growth factors such as transforming growth factor-beta (TGF-β) and insulin-like growth factor-1 (IGF-1), particularly in regions with a high aggrecan-fixed charge density. The limited regenerative capacity of cartilage is closely associated with matrix stiffness and the localized presence of bioactive growth factors within the agrecan-rich ECM. In highly compressed areas, Na^+^ concentrations may exceed a critical threshold, triggering the release and activation of growth factors sequestered within the ECM. This Na^+^-dependent mechanism is pivotal for cartilage maintenance and repair in response to mechanical loading. Thus, mechanical loading directly influences the sodium concentration, which ranges from 250–400 mM—nearly three times that of most other tissues—thereby supporting the regulated release of growth factors necessary for preserving cartilage structure and function [57]. However, in osteoarthritic cartilage, the degradation of aggrecan leads to a reduction in fixed charge density and Na^+^ levels, compromising this regulatory pathway and weakening the integrity of cartilage [60]. These findings highlight the critical role of inorganic ions, many of which are derived from dietary micronutrients and present in synovial fluid, in facilitating the displacement and activation of growth factors—including both systemic growth hormones and local intra-articular factors—underscoring their critical function in the load-dependent maintenance of cartilage, particularly through the preservation of aggrecan integrity.

In early-stage OA, the structural and biomechanical integrity of the hyaline cartilage is progressively compromised, triggering a cascade of degenerative changes [10,32,62]. One of the earliest alterations involves the enzymatic degradation of the proteoglycan-rich ECM, specifically aggrecan, leading to a reduction in fixed charge density and osmotic swelling pressure. This loss of proteoglycan content impairs the ability of the cartilage to attract and retain water, critically reducing its compressive resilience and viscoelastic properties. As aggrecan degradation progresses, the integrity of the collagen fibrillar network deteriorates, particularly in the superficial and deep zones, where tensile strength is reduced and resistance to shear and compressive forces is impaired. Disruption of the interaction between collagen and proteoglycans further reduces the capacity of the cartilage to distribute mechanical loads uniformly, resulting in increased focal stress and accelerated tissue damage. Biomechanically, the degradation of the arcade-like collagen fiber organization weakens the anchorage of cartilage to the subchondral bone, destabilizing the cartilage–bone interface and increasing the risk of mechanical failure [21,23,26,41,48,63]. Simultaneously, disturbances in ionic homeostasis, including reduced sodium and calcium regulation, disrupt the pericellular matrix [62]. These changes diminish chondrocyte viability, compromise their ability to detect and respond to mechanical stimuli, and hinder mechanotransduction and repair mechanisms, further exacerbating matrix degradation [53,54,55,56,57,58,59,60,61]. These interconnected compositional, structural, and biomechanical alterations collectively contribute to the hallmark features of OA, such as increased cartilage stiffness, decreased elasticity, and the development of fibrillations and surface erosions. Over time, persistent low-grade inflammation, ECM degradation, and chondrocyte terminal differentiation (hypertrophy) [10] drive additional pathological changes, including subchondral bone sclerosis and endochondral ossification [46,63], ultimately leading to joint failure. These degenerative processes exacerbate joint dysfunction, underscoring the critical importance of therapeutic strategies aimed at preserving aggrecan integrity [64,65,66,67,68], protecting the collagen network, and maintaining the intricate biomechanical equilibrium of cartilage to halt or slow OA progression.

## 3. Aggrecan Degradation and Its Central Role in Cartilage Stability, Resilience, and Protection Against Early OA

**Figure 2:**
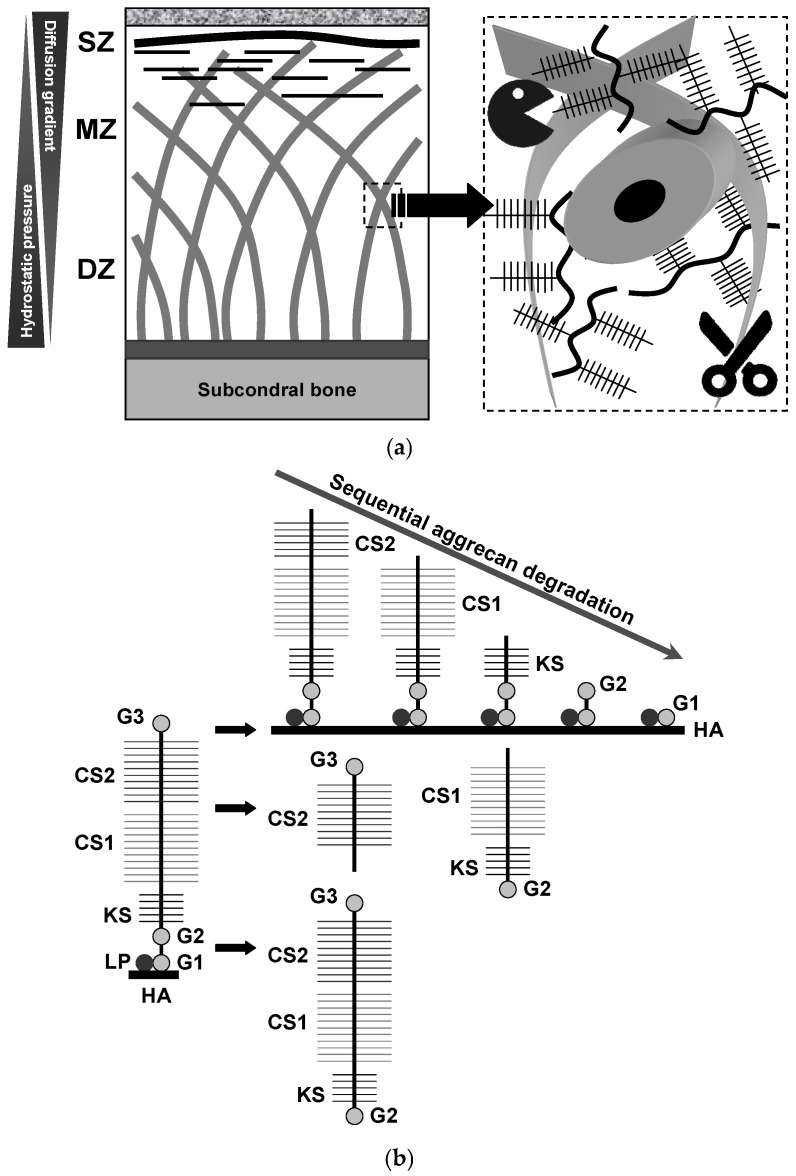
Schematic illustration of cartilage degradation and aggrecan cleavage pathways. (**a**) Overview of cartilage pathophysiology, focusing on the loss of ECM integrity due to the enzymatic breakdown of aggrecan and collagen. These components are critical for maintaining cartilage elasticity, compressive resistance, and structural integrity, and their degradation underlies tissue degeneration in conditions such as OA. (**b**) Enzymatic cleavage of aggrecan by ADAMTS and MMPs. Cleavage of the G1 and G2 domains disrupts the binding of aggrecan to hyaluronan, weakening matrix cohesion. Additional cleavage between the CS1 and CS2 domains depletes the polysulfated glycosaminoglycan chains, reducing water retention and the ability of hyaline cartilage to resist compressive forces. Sequential degradation from the G3 domain leads to the progressive disassembly of the aggrecan molecule. Simultaneously, hyaluronan fragmentation due to mechanical stress or oxidative damage generates oligosaccharides that influence inflammatory pathways and cellular signaling, exacerbating ECM breakdown and driving cartilage degeneration. This degradation compromises the biomechanical properties of cartilage, ultimately affecting joint health. Abbreviations: SZ, superficial zone; MZ, middle zone; DZ, deep zone; HA, hyaluronic acid (hyaluronan); LP, link protein; CS1 and CS2, chondroitin sulfate-rich domains of aggrecan; KS, keratan sulfate-rich region; G1, G2, and G3, globular protein domains of aggrecan.

Aggrecan catabolism, driven by elevated pro-inflammatory cytokines and matrix-degrading enzymes, is a hallmark of cartilage damage during OA development [10,68,69,70,71,72,73] with distinct patterns observed across different endotypes of the disease. Pratta et al. [71] demonstrated that aggrecan plays a protective role in maintaining cartilage integrity by impeding collagen degradation through interference with the activity of collagenases responsible for fibril cleavage. Therapeutic strategies aimed at inhibiting aggrecanolysis show promise in delaying or preventing early cartilage degradation [73]. These findings underscore the potential of selective aggrecanase inhibitors to simultaneously preserve aggrecan and type II collagen, providing comprehensive protection for joint cartilage. By maintaining interactions with collagen fibrils, SLRPs are thought to stabilize the fibrillar network, mitigating extensive proteolytic damage during the early stages of cartilage degeneration [27,31,32,33,34,35,36,37,38,39,74]. Unlike aggrecan, which exhibits notable susceptibility, SLRPs demonstrate significant resistance to proteolytic cleavage and release during culture [75]. This evidence suggests that SLRPs, including decorin, biglycan, and lumican, are less prone to proteolytic degradation during interleukin-1 (IL-1)-induced cartilage catabolism compared to aggrecan. These observations highlight the pivotal role of aggrecan degradation in the early stages of OA, indicating that it may be the “weakest link” to cartilage integrity (Figure 2). Therefore, preventing aggrecanolysis is critical for maintaining cartilage homeostasis and halting progression in early OA. Furthermore, since collagenase activation is a key driver in the transition from early-stage OA to established (chronic) OA [72], timely interventions that target both collagenolysis and aggrecan breakdown are essential for halting the early cycle of joint degeneration.

The question of whether targeting a single catabolic mediator, such as tumor necrosis factor-alpha (TNF-α), interleukin-1 beta (IL-1β), matrix metalloproteinases (MMPs), or a disintegrin and metalloproteinase with thrombospondin motifs (ADAMTS) enzymes, is sufficient to halt cartilage degradation during OA development and progression remains uncertain [66]. Matrix degradation products, including COMP and fibronectin proteolytic fragments (FN-fs), can trigger low-grade inflammation through feedback loops, suggesting that chondroprotective agents may potentially disrupt this cycle. Among these agents, glucosamine has been widely studied for its anti-catabolic and pro-anabolic effects in various in vitro models [76,77,78,79,80,81,82,83,84,85]. It has been shown to enhance the synthesis of proteoglycans, including hyaluronic acid and sulfated glycosaminoglycans, while inhibiting their enzymatic degradation by suppressing the expression of matrix metalloproteinases [76,77,78,79] and pro-inflammatory cytokines [79,80,81,82,83]. Piepoli et al. [81] demonstrated that glucosamine sulfate effectively inhibits IL-1β-induced gene expression and reduces the production of pro-inflammatory cytokines in cultured chondrocytes. Similarly, Dodge et al. [76] found that glucosamine sulfate (1 µM) significantly decreases the production of MMP-3 in chondrocytes. These findings, along with those of numerous others, indicate that glucosamine modulates multiple mechanisms to counteract both inflammatory and catabolic processes, underscoring its potential as a therapeutic agent for protecting cartilage integrity in early-stage OA. This ability to alter the course of the disease supports its classification as a disease-modifying osteoarthritis drug (DMOAD).

Nevertheless, the role of chondroprotective agents, such as glucosamine and chondroitin sulfate, in OA management has been a subject of considerable debate, with clinical evidence presenting contrasting perspectives on their efficacy. Various factors, including differences in salt types, biases in study design, and other confounding variables, may explain some of the inconsistencies in the evidence [9,14]. Some studies suggest that these agents may offer symptomatic relief by slowing the progression of OA and improving joint function, particularly in the early stages of the disease. For example, a meta-analysis by Zhu et al. [8] indicated that glucosamine and chondroitin sulfate may provide moderate improvements in pain and function in knee OA patients. However, other clinical trials have failed to demonstrate significant benefits compared with placebos, leading to skepticism regarding their disease-modifying potential. A notable example is the GAIT trial [86,87,88], which found minimal improvements in pain and function with glucosamine and chondroitin sulfate in patients with knee OA, particularly in those with moderate to severe disease. This discrepancy highlights the need for further investigation into the mechanisms underlying their effects, as well as the identification of patient subgroups that may benefit the most from these treatments. In particular, focusing on early-stage OA endotypes rather than the traditional emphasis on end-stage OA phenotypes could provide more targeted insights in the context of patient stratification [89,90]. Despite controversial evidence, the persistence of glucosamine and chondroitin sulfate in clinical practice reflects a demand for more effective disease-modifying therapies for OA, underscoring the importance of exploring novel approaches that may complement or enhance their effects. A recent study introduced the concept that PALs can influence the diffusion and effectiveness of these agents, potentially explaining the variability in treatment outcomes [17]. This evolving understanding underscores the complexity of OA management and emphasizes the need for more targeted and individualized approaches in clinical practice.

Disrupting the chronic cycle of cartilage matrix degradation and joint inflammation may require a combination of proteinase inhibitors and agents targeting specific pathways involved in cartilage degeneration [10]. FN-fs play a critical role in exacerbating cartilage damage by inducing chondrolysis and proteoglycan depletion through the generation of reactive oxygen species (ROS)—a key driver of cartilage degeneration—and activation of catabolic cytokines. Antioxidants such as N-acetylcysteine have been shown to effectively counteract these effects by significantly reducing FN-fs-mediated cartilage chondrolysis [69,70]. N-acetylcysteine neutralizes ROS, inhibits FN-fs-induced proteoglycan loss, and promotes aggrecan restoration by suppressing catabolic cytokine activity [70]. Collectively, these findings highlight the potential of glucosamine sulfate and N-acetylcysteine as therapeutic agents for reversing the early cartilage damage mediated by FN-fs, ROS, and inflammatory cytokines. Future research should focus on elucidating the precise mechanisms through which N-acetylcysteine and other inhibitors function, which could lead to the development of novel therapeutic compounds that specifically target matrix-derived peptides, cytokines, and proteolytic enzymes such as ADAMTS and MMPs. Addressing challenges in efficient drug delivery, including the precise targeting of cartilage tissue and ensuring sustained release, will be crucial for translating these findings into effective therapies. Optimizing the bioavailability of these compounds could significantly enhance their effectiveness in preventing cartilage degeneration, thereby improving clinical outcomes [17].

## 4. Mechanisms of Solute Transport in Hyaline Cartilage: Insights into Synovial Fluid Dynamics, ECM Barriers, and the Role of Joint Motion

Articular cartilage is a unique connective tissue that is avascular, aneural, and alymphatic and relies on diffusion and convection to transport essential nutrients, metabolites, and signaling molecules, thereby supporting cellular health and maintaining tissue homeostasis [22]. The cartilage ECM plays a critical role in determining the transport properties of the tissue. Composed of collagen fibers, proteoglycans, and glycosaminoglycans, the ECM forms a dense network that not only provides structural integrity but also selectively regulates the movement of solutes [91]. The negative charge of glycosaminoglycans creates an electrochemical environment that influences the transport of charged molecules, whereas the collagen network contributes to the tensile strength of the tissue and restricts molecular movement by limiting the pore size within the matrix. Small solutes, such as oxygen, glucose, and ions, diffuse readily through the ECM, while larger molecules, including growth factors, cytokines, and enzymes, face hindered transport owing to steric exclusion and electrostatic interactions [30,92]. Diffusion, a passive process driven by concentration gradients, is the primary mechanism for the movement of small solutes, whereas convection, which is enhanced by mechanical loading, facilitates the transport of larger molecules within the matrix. As shown in Table 1, this distinction is particularly significant for drug delivery, as small therapeutic agents, such as glucosamine sulfate, rely on diffusion, whereas larger agents require mechanical forces, such as compression, to overcome the restrictions of the ECM and achieve deeper tissue penetration. Understanding solute transport mechanisms is crucial for optimizing strategies aimed at improving cartilage repair and providing targeted drug delivery.

A key factor influencing solute movement within articular cartilage is the liquid barrier formed by synovial fluid, which generates a stagnant film or an unstirred layer at the cartilage surface. This phenomenon was first reported by Maroudas et al. [30] and has been further supported by more recent studies, which demonstrated the presence of this unstirred layer at the solid–liquid interface [93], where solutes accumulate in stagnant fluid due to minimal motion. The findings from these investigations—initially conducted using classic dye solution experiments with methylene blue (MW: approx. 320 Da) as a probe molecule [30] and later using iodixanol (MW: 1560 Da) as a neutral contrast agent in combination with microcomputed tomography and biphasic-solute finite element models [93]—demonstrated that unstirred layers play a significant role in modulating the diffusion of both small and medium-sized solutes. Furthermore, pioneering research on solute permeation through planar bilayer lipid membranes has shown that smaller molecules with higher diffusion coefficients experience a thicker unstirred layer than larger molecules with lower diffusion coefficients [94]. The thickness and concentration of this stagnant liquid film are strongly influenced by the stirring rate, especially in viscous biological fluids such as the synovial liquid found in larger diarthrodial joints. Under conditions of minimal movement, the fluid barrier significantly hinders passive diffusion, making the rate of solute transport into the cartilage highly dependent on joint motion. Conversely, increasing the stirring rate—through joint articulation—helps to disrupt these stagnant layers, thus enhancing solute transport into the cartilage matrix.

Once solutes pass through the synovial fluid barrier, they encounter a dense ECM, which further restricts their movement based on factors such as size, charge, and molecular interactions. As previously mentioned, the ECM is composed of a network of collagen fibers and proteoglycans that together form a highly hydrated matrix. Its permeability is influenced by cartilage deformation, with pore sizes ranging from 2.5 nm to 6.5 nm [91]. These small pore sizes limit the penetration of larger solutes such as growth factors, cytokines, and matrix degradation products. However, permeability varies across the cartilage. Superficial regions near the joint surface exhibit higher permeability owing to lower collagen and proteoglycan content, whereas deeper regions, which are richer in ECM components, have lower permeability. Mechanical compression of cartilage further decreases permeability by limiting the flow of interstitial fluid and solute transport [55,91]. Interestingly, physical stimuli—particularly cyclic loading—can enhance the transport of larger molecules by increasing ECM permeability and facilitating fluid movement [92]. The authors identified a fundamental mechanism underlying the dynamic “pumping effect” induced by repetitive mechanical loading, which is essential for the transport of high-molecular-weight molecules. Their work demonstrated that cyclic loading significantly enhanced the transport efficiency of large molecules such as serum albumin (MW: 66.5 kDa) [92]. Using human femoral head cartilage plugs immersed in a medium containing radioactive solutes, such as [14C] urea (MW: ~60 Da) and [125I] human serum albumin (MW: 66.5 kDa), and subjected to simulated walking cycles with pressures ranging from 2 to 8 Mpa at a frequency of 1 Hz, the authors observed in the same study a 30–100% increase in the transport rate of large solutes in loaded cartilage compared to static conditions [92]. This finding underscores the critical role of mechanical loading in overcoming the diffusion limitations posed by the dense ECM of cartilage, particularly for macromolecules such as COMP (MW: ~200 kDa), FN fragments (MW: 50–200 kDa), and cytokines (MW: 8–60 kDa). Although mechanical loading does not significantly affect the movement of small solutes, such as glucose and oxygen, it is essential to facilitate the transport of macromolecules [92]. This process of fluid “pumping”, induced by repetitive mechanical loading, not only promotes the transport of large solutes into the cartilage, including newly synthesized matrix macromolecules, but also plays a critical role in the removal of matrix degradation products into the synovial fluid, thereby supporting cartilage health.

**Table 1:** Physicochemical properties and transport behavior of solutes in cartilage.

Physicochemical Property	Small Solutes *	Medium-Sized Solutes **	Large Solutes ***
Molecular weight, (MW, Da)	<500	500–50,000	>50,000–1,000,000
Hydrodynamic radius (nm)	<1.0	1.0–10	10–200
Charge characteristics	Neutral to slightly negative	Weakly charged	Highly charged
Diffusion coefficient (cm^2^/s)	10^−5^–10^−7^	10^−8^–10^−11^	10^−12^–10^−13^
Synovial residence time (half-life, t½)	Minutes to hours	Hours to days	Days to weeks
Cartilage penetration (no unstirred layers)	High—Rapid diffusion, enhanced by synovial fluid agitation and improved access to the ECM	Moderate—Partial penetration, further enhanced by effective mixing and reducing unstirred layers	Low—Generally restricted by the dense ECM; agitation may slightly improve penetration but remains limited
Transport mechanism	Diffusion	Diffusion and convection	Convection, surface adsorption, the limited diffusion
Targeting strategies for optimized delivery in response to joint motion (sliding vs. compression)	Sliding: Significantly enhances diffusion and surface uptake. Compression: Minimal effect, with slight clearance increase	Sliding: Enhances synovial mixing, promoting transport into cartilage. Compression: Facilitates penetration of medium-sized solutes into deeper layers	Sliding: Promotes surface adsorption and increases retention time. Compression: Increases retention within ECM due to pressurization, enhancing deep penetration of solutes

* Small Solutes: include nutrients (glucose, amino acids and oxygen), metabolic waste byproducts (CO_2_ and lactate), small DMOADs (e.g., glucosamine sulfate), and antioxidants (e.g., N-acetylcysteine). ** Medium-Sized Solutes: Cytokines (e.g., TNF-α and IL-1β) and fibronectin fragments. *** Large Solutes: Matrix degradation products (e.g., COMP and fibronectin fragments), hyaluronic acid, serum albumin, growth factors (e.g., IGFs and TGF) and enzyme inhibitors (e.g., ADAMTS and MMPs). The table includes data from studies [30,91,92,94,95,96,97,98,99,100,101,102,103,104,105,106,107] based on Fick’s first law of diffusion (describing solute movement along concentration gradients), the Stokes–Einstein equation (linking diffusion coefficients to molecular size and medium viscosity), and Darcy’s law (governing fluid flow in porous media such as cartilage and relating permeability to solute transport).

Beyond the well-established mechanisms of solute transport in articular cartilage, recent research has highlighted the significant role of sliding motions in enhancing substance movement [102,107]. These studies have shown that sliding motions dramatically accelerate molecular transport into the cartilage surface, far exceeding the uptake observed with compression or diffusion alone. Using fluorescent solute tracking and confocal microscopy, researchers found that sliding motions lead to solute uptake rates several orders of magnitude higher than those achieved through pressurization or passive transport. In particular, Graham et al. [102] reported that sliding at a speed of 60 mm/s resulted in substantial solute transport into the buried contact area of the cartilage, an effect that was not observed at slower speeds. These findings underscore the crucial role of joint movement—particularly sliding—in facilitating efficient small-solute diffusion within the cartilage matrix. Additionally, Culliton et al. [107] confirmed that sliding is significantly more effective than axial compression in promoting solute uptake, reporting a 2.1-fold increase after 30 min of sliding and a 4.4-fold increase after 2 h compared with compression. The primary mechanism underlying this enhanced uptake is the generation of hydrodynamic pressure between articulating surfaces, which drives advective flow and pushes solutes deeper into the cartilage matrix. This process is far more effective than passive diffusion or axial compression in delivering nutrients and clearing waste products, particularly in deeper regions that would otherwise be poorly accessed by diffusion alone [102,107]. These insights have significant implications for maintaining cartilage health and developing therapeutic strategies for OA and other degenerative joint conditions.

On the basis of the elegant biphasic theory developed by Mow et al. [91], solute transport in articular cartilage is a complex process influenced by the interaction of mechanical forces and the poroviscoelastic properties of the ECM. As summarized in Table 1, small solutes benefit from the enhanced mixing of synovial fluid during joint movement, particularly during sliding motions [102,107], which disrupt stagnant layers and facilitate diffusion. In contrast, larger solutes, such as growth factors and cytokines, rely on mechanical loading to overcome the diffusion barriers posed by the ECM [92]. Understanding these solute transport mechanisms is essential for optimizing therapeutic approaches, including the development of chondroprotective agents, to maintain cartilage health and address cartilage-related diseases. The novel sliding-induced solute transport mechanism offers a promising strategy for improving drug delivery and cartilage repair, particularly in the early stages of OA. By leveraging joint dynamics such as sliding motions, these findings open up new opportunities for optimizing nutrient delivery, waste removal, and therapeutic interventions for cartilage diseases.

## 5. Joints in Rhythm: Circadian Regulation and Its Influence on Cartilage Health and OA Pathogenesis via the Molecular Clock

Circadian rhythms are intrinsic, roughly 24 h light/dark cycles in biological processes that are driven by a core set of clock genes and their protein products [108]. In mammals, these include the circadian locomotor output cycle kaput (CLOCK), brain and muscle ARNT-like protein1 (BMAL1), dimerize and bind to E-box promoter elements of the transcriptional repressor period (Per1 and Per2), and crypto-chrome (Cry1 and Cry2). These genes form a transcriptional–translational feedback loop that orchestrates various cellular functions. In hyaline cartilage, the circadian regulation of anabolic and catabolic genes likely synchronizes cartilage repair and remodeling, leading to their occurrence at optimal times of the day and enhancing tissue maintenance and function. Several studies have demonstrated the expression of these clock genes in chondrocytes [18,20,109,110,111,112,113,114,115,116,117,118,119,120,121,122,123,124,125], indicating that these primary cell types in cartilage possess autonomous time-keeping circadian clocks that allow them to predict and adapt to environmental factors. Chondrocytes exhibit daily variations in gene expression and activity, which align with the overall circadian rhythm of the body. The circadian clock regulates the expression of ECM-related genes, including those encoding aggrecan, collagen, and other matrix glycoproteins. Despite the complexity and novelty of chronobiology and circadian rhythms in cartilage, recent reviews have sought to systematically organize the limited data available [19,112,118,126,127,128,129,130]. Emerging evidence suggests that chondrocyte circadian rhythms regulate several key functions, including matrix synthesis and degradation, cellular metabolism, and the response to mechanical stress. For instance, the expression of MMPs and ADAMTS, enzymes involved in cartilage matrix turnover, has been shown to follow a circadian pattern [130]. This rhythmic activity suggests temporal regulation of cartilage maintenance and repair, potentially optimizing tissue function and integrity in response to daily fluctuations in joint loading and other environmental factors.

The circadian clock In hyaline cartilage influences both diurnal and nocturnal variations in cellular activity [20]. During the day, chondrocytes are typically more active in synthesizing ECM components such as proteoglycans and non-collagenous proteins, which are crucial for maintaining cartilage structure and function. At night, metabolic processes shift towards glucose metabolism, providing energy for cellular processes, while enzymes such as MMPs and aggrecanases are upregulated to promote ECM turnover and remodeling. This diurnal and nocturnal variation ensures a balance between cartilage repair and degradation, optimizing tissue resilience and function in response to daily cycles of joint loading and unloading [20,125,131,132]. Circadian regulation at various stages may underlie the observed daily fluctuations in protein abundance. Several studies have highlighted the importance of circadian rhythms in cartilage. Gossan et al. [18] identified disruptions in clock gene expression in experimental mouse models, suggesting a critical role of circadian rhythm disturbances in the early stages of OA pathogenesis. These findings highlight that intrinsic circadian clocks in murine chondrocytes are essential for cartilage homeostasis and their disruption may significantly contribute to the development and progression of OA. In addition, Dudek et al. [20] demonstrated that cartilage physiology is governed by distinct circadian patterns, with proteasomal degradation peaking in the morning, protein synthesis in the afternoon, ATP production in the evening, and glucose metabolism at night. These findings emphasize the critical role of circadian regulation in the temporal coordination of metabolic activities within the cartilage [20]. An anabolic peak also occurs at night, coinciding with the resting phase, which may facilitate cartilage recovery and ECM synthesis following daily mechanical stress [18]. Additionally, Yang et al. [19] reported that anabolic ECM genes, including fibrillins, laminins, and netrin, exhibited peak expression in the early morning, along with catabolic genes that control proteolysis. This highlights the complexity of circadian regulation in cartilage and suggests that different types of metabolic and structural processes may follow distinct temporal patterns. Importantly, the timing of cartilage repair and destruction underscores the essential role of circadian rhythms in maintaining tissue homeostasis.

Circadian regulation of ECM genes plays a vital role in cartilage homeostasis, and rhythmic gene expression ensures proper coordination of ECM synthesis, remodeling, and degradation in alignment with daily physiological cycles [18,20,111,120,132,133]. These mechanisms support cartilage repair at rest and function during mechanical loading, facilitated by the strategic release of growth factors stored in the ECM in response to mechanical and osmotic changes [132]. This process enhances chondrocyte activity, stimulates extracellular matrix (ECM) synthesis, and maintains tissue integrity. Recent studies highlight FoxO1 as a key regulator of both ECM integrity and circadian rhythm genes in chondrocytes [120,133]. Disruption of circadian rhythms reduces anabolic activity while promoting catabolic processes, including upregulation of matrix-degrading enzymes, which accelerate cartilage degeneration. FoxO1 involvement in the TGF-β/TAK1 signaling pathway, a known protective mechanism in OA, may also be impaired under circadian misalignment, further contributing to disease progression [120]. Furthermore, pro-inflammatory cytokines, such as IL-1, disrupt chondrocyte circadian clocks by altering core clock gene expression, leading to dysregulation of anabolic and catabolic processes and increasing matrix degradation [111].

Mechanical loading and hyperosmolarity also play essential roles in regulating circadian rhythms in the cartilage [132]. Pioneering studies [56] demonstrated that compression-induced osmotic pressure changes release matrix-bound growth factors, such as TGF-β and IGF, which stimulate chondrocyte metabolism and ECM production. This release enhances cartilage function and resilience, with hyperosmolarity further activating the signaling pathways that regulate chondrocyte proliferation, differentiation, and matrix repair [132]. These biomechanical forces and osmotic fluctuations also synchronize chondrocyte activity with circadian rhythms, optimizing the timing of repair and maintenance processes. Compression-driven fluid displacement and localized osmotic shifts further enhance chondrocyte activity by altering ion concentrations, triggering metabolic responses, and promoting cartilage repair [56]. These findings highlight the interplay between biomechanical forces, osmotic regulation, and circadian rhythms, emphasizing their critical role in cartilage homeostasis. Therefore, preserving natural circadian rhythms may be a key strategy for preventing cartilage-related diseases, such as OA.

In addition, circadian rhythms are critical for chondrocyte function, ensuring that metabolic processes in the articular cartilage are synchronized with daily cycles of activity and repair. Dudek et al. [20] demonstrated that the circadian clock orchestrates key cellular processes, enhancing metabolic efficiency and promoting tissue regeneration during periods of physical activity. Since glycolysis is the primary ATP source for chondrocytes [134,135,136], the circadian regulation of glucose transporters such as Slc2a1 (GLUT1) and glycolytic enzymes such as pyruvate kinase (PKM) are critical for maintaining energy balance. These metabolic regulators exhibit rhythmic expression at both protein and mRNA levels, demonstrating precise adaptation to the 24 h cycle [20], thereby enabling cartilage to adapt effectively to the oxygen gradient, which ranges from approximately 6% O_2_ in the superficial zone to less than 1% O_2_ in the deep zone [137,138,139,140]. This adaptation supports the metabolic shift from oxidative phosphorylation to anaerobic glycolysis [136], which is essential for cartilage homeostasis under hypoxic conditions.

Hypoxia-inducible factors (HIFs), which are critical transcription factors in hypoxic environments, regulate energy metabolism, matrix synthesis, and cell survival in chondrocytes [141,142]. Although HIFs drive essential metabolic adaptations under hypoxic conditions, excessive or prolonged activation can disrupt the circadian rhythm, leading to compromised cartilage function. Stabilizing HIF-1α has been shown to reduce hypoxia-induced apoptosis, senescence, and matrix degradation while promoting collagen synthesis and maturation. This results in a denser, more resilient collagen matrix that protects against cartilage breakdown, offering a potential mechanism for the prevention of early OA progression [141].

However, modern sedentary lifestyles, particularly those associated with nutrient deprivation in thicker cartilage, such as those found in the human knee and hip joints [90], may significantly counteract these protective mechanisms by exacerbating mitochondrial dysfunction, a known contributor to ROS accumulation and cellular stress. The overproduction of ROS triggers oxidative stress, impairs chondrocyte function, and accelerates ECM degradation [137,139,143,144]. The combined effects of circadian misalignment, hypoxia, and metabolic stress can exacerbate these processes significantly. Targeting these interconnected pathways—by restoring circadian alignment and mitigating hypoxia and oxidative stress—offers promising opportunities for preventing early cartilage damage and improving outcomes in individuals at risk of early-stage OA.

Taken together, the discovery of circadian rhythms in hyaline cartilage challenges the traditional view of cartilage as a passive structure and transforms our understanding of joint biology by emphasizing its dynamic and time-regulated role in maintaining joint health and function. These intrinsic rhythms synchronize the key processes of cartilage repair and degradation, underscoring their critical role in tissue homeostasis and resilience. Exploring the underlying mechanisms of these temporal dynamics could unlock novel approaches for preventing and managing OA. Addressing circadian pathways may enable the design of innovative therapies that align with the natural timing of cartilage maintenance, thereby enhancing their effectiveness while minimizing adverse effects. As emerging evidence increasingly highlights the importance of chronobiology in joint health, the following section aims to further investigate this promising field and explore its transformative potential in advancing next-generation therapies to combat OA and other degenerative joint disorders.

## 6. Timing Is Everything: Synchronizing Chondroprotective Therapy with Circadian Rhythms and Physical Activity/Exercise for Optimal Cartilage Health

Circadian rhythms and physical activity play pivotal roles in regulating cartilage metabolism and joint health, providing a foundation for innovative approaches to optimizing chondroprotective therapies [17,20]. Cartilage undergoes rhythmic fluctuations in anabolic and catabolic activities governed by intrinsic biological clocks and external factors such as mechanical loading. These cycles establish critical windows for therapeutic intervention, where aligning the timing of drug delivery and targeted physical exercise programs with the natural rhythms of cartilage can significantly enhance outcomes. Emerging evidence also indicates that the bioavailability of commonly used chondroprotective agents within cartilage tissue may be limited by diffusion barriers such as synovial liquid and the ECM [17,90,105]. Joint motion promotes the production of synovial fluid components, such as hyaluronic acid, and facilitates nutrient and drug transport to the cartilage, reinforcing the value of synchronized treatments [30,92,101,145,146]. To fully unlock this potential, it is crucial to refine drug delivery strategies to enable deeper cartilage penetration and prolonged retention at the target site [17], thus addressing both spatial and temporal challenges. By integrating the cutting-edge knowledge of cartilage biology, circadian rhythms, the effects of physical activity, and targeted intra-cartilage drug delivery, we can develop a comprehensive strategy that overcomes the limitations of traditional therapies and promotes optimal cartilage repair and function. This section explores how synchronizing circadian medicine with biochemical and mechanical factors can reveal new possibilities for preserving cartilage health and mitigating OA development and progression.

### 6.1. Targeting Ultra-Early-Stage OA: Unlocking the Potential of Optimizing Chondroprotective Strategies

The quest for effective disease-modifying osteoarthritis drugs (DMOADs) has been a major focus of research in recent years, yet outcomes have frequently failed to meet expectations [6]. One significant factor contributing to these disappointments is the stage of OA at which most studies have been conducted. Emerging evidence suggests that targeting the early subclinical stages of OA—characterized by molecular and cellular disruptions preceding radiographic and/or symptomatic damage—may offer a critical window for effective interventions [9]. Moreover, multimodal approaches that combine chondroprotective drugs with complementary therapies have shown promise in enhancing therapeutic outcomes in OA management. In this context, early administration of chondroprotective agents, such as glucosamine and chondroitin sulfate, has been shown to provide substantial benefits, particularly before significant disease progression [9]. However, current research and clinical trials primarily target established OA, a symptomatic clinical stage in which joint damage is typically severe and may already be irreversible. This late-stage focus inherently limits the potential impact of pharmacological intervention. In contrast, the early stages of OA remain largely neglected, despite their significant therapeutic potential. These early subclinical changes represent a pivotal moment for intervention, offering an opportunity to modify the disease course and potentially prevent progression to debilitating stages. Redirecting research efforts to these early stages could enable the development of treatments capable of modifying the disease trajectory, slowing progression, and potentially preventing OA from progressing to debilitating stages. Such a paradigm shift has the potential to transform OA management by reducing the prevalence of symptomatic disease and alleviating its substantial public health burden. Further research on the efficacy of chondroprotective agents in these early stages is urgently needed, as it may reveal innovative strategies for early detection and intervention. Ultimately, advancing our understanding of early-stage OA could pave the way for more effective therapeutic approaches, promote long-term joint health, and significantly improve patient outcomes.

### 6.2. Targeted Aggrecanolysis Inhibition as a Promising Approach to Preventing Cartilage Breakdown

The sequential roles of ADAMTS-4 and ADAMTS-5 [64,147,148,149] and MMP-13 and MMP-14 [65,67,150,151,152,153,154] in cartilage matrix degradation illustrate the complex, stage-specific progression of early OA. During the initial stages, the enzymatic activities of ADAMTS-4 and ADAMTS-5 drive the cleavage of aggrecan. This proteoglycan loss results in the depletion of cartilage proteoglycans, reducing their compressive strength, and predisposing the matrix to further mechanical and biochemical insults. As OA progresses, there is a pathological shift toward MMP-mediated degradation, where MMP-13 and MMP-14 become predominant, targeting collagen fibers that are essential for the tensile strength of the cartilage. This sequential enzymatic degradation highlights the need for tailored stage-specific therapeutic strategies. Inhibiting aggrecanases during the early stages of OA could preserve proteoglycan content and maintain cartilage integrity, whereas targeting collagenases at later stages may help mitigate irreversible damage. Collagen breakdown represents a “tipping point” in OA progression, as its damage is largely irreversible and leads to profound structural and functional impairments [155]. Furthermore, collagen has an exceptionally slow turnover rate with a half-life exceeding 100 years, rendering accumulated damage permanent [156]. Understanding these processes is critical for the effective targeting of early cartilage breakdown. Aligning treatments with phase-specific enzyme activity offers a promising approach for improving the efficacy of early-stage OA interventions and slowing disease progression.

### 6.3. Chronobiology and DMOAD Drug Delivery: Enhancing Efficacy in OA Management

A fundamental question in cartilage biology is how chondrocytes develop their remarkable capacity to maintain homeostasis and withstand the daily biomechanical stresses associated with cycles of rest and activity. Recent studies have shown a functional circadian clock within ECM-rich hyaline cartilage, which is crucial for coordinating tissue homeostasis [18,19,20,125,131,132,141,157].

As mentioned earlier, this temporal regulation of the ECM and associated metabolic processes is believed to provide a protective mechanism, enabling cartilage to efficiently adapt to the rhythmic demands of daily activities. Alterations in the microenvironment of chondrocytes, such as decreased glucose and oxygen levels, increased anaerobic metabolism, and lower pH, can disrupt the circadian clock [141,157]. The intricate connection between the hypoxia response system and the circadian clock highlights the complex interplay of metabolic and temporal regulation in maintaining cellular homeostasis. In deeper zones of cartilage, where nutrient diffusion is limited [90], chondrocytes may adopt a secretory phenotype characterized by the production of ROS and inflammatory cytokines such as TNF-α, IL-6, and IL-1, which are key mediators of matrix degradation [158,159,160,161,162]. This phenotypic shift can lead to the activation of proteolytic enzymes, including ADAMTSs and MMPs, which degrade ECM, particularly aggrecan. Consequently, these processes contribute to cartilage degradation and play a significant role in OA onset. In sedentary individuals, disruptions in circadian rhythms may further compromise this regulatory mechanism, increasing the risk of OA development and progression. Overall, evidence strongly supports the notion that mitochondrial bioenergetic dysfunction in cartilage plays a critical role in the metabolic imbalance that transitions from healthy to critical ultra-early OA states.

**Figure 3:**
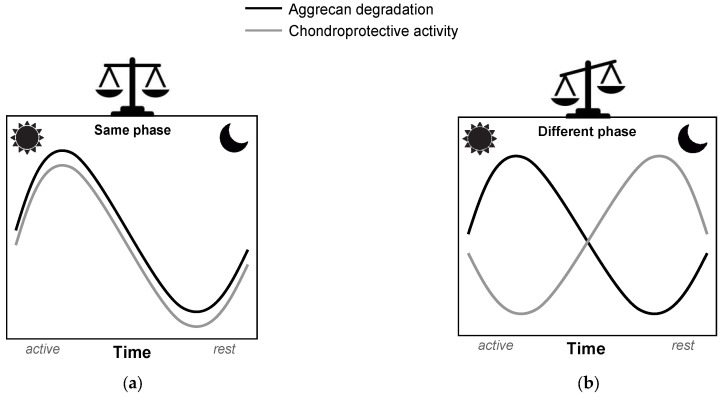
Synchronizing chondroprotective therapy with circadian timing and cartilage metabolism: same-phase versus different-phase strategies. (**a**) Schematic representation of the “same-phase” approach, in which an oral chondroprotective agent is administered in synchronization with the early morning peak of cartilage catabolic activity. The sinusoidal curve represents the diurnal variation in cartilage matrix degradation, with the highest point indicating the peak of catabolic processes. Administering a disease-modifying agent during this peak period maximizes its therapeutic effects by directly counteracting matrix degradation when it is most pronounced, thereby enhancing cartilage preservation. (**b**) Diagram illustrating the “different-phase” approach, in which the chondroprotective agent is administered orally in the evening, misaligned with the morning peak catabolic activity. The sinusoidal curve highlights the temporal mismatch, indicating that the bioavailability of the drug does not coincide with the peak cartilage breakdown phase, potentially resulting in reduced therapeutic efficacy. These schematic illustrations emphasize the critical importance of aligning chondroprotective therapy with the circadian rhythm of cartilage metabolism. Optimal timing of drug administration, synchronized with peak degradation periods, offers a more effective strategy for slowing cartilage breakdown and potentially preventing early-stage OA progression.

The circadian rhythm of cartilage metabolism offers a critical framework for optimizing the timing and efficacy of chondroprotective therapies during the early stages of OA. Cartilage exhibits rhythmic fluctuations in metabolic activity, with matrix degradation peaking in the early morning [20], driven by elevated levels of matrix-degrading enzymes such as ADAMTS and MMPs, inflammatory cytokines such as TNF-α and IL-1β, and matrix degradation products such as COMP and FN fragments. This catabolic peak represents a window of increased cartilage vulnerability, making it an optimal target for therapeutic interventions. Based on these findings, Figure 3a illustrates a “same-phase” approach, where the administration of chondroprotective agents is synchronized with this morning catabolic peak, allowing the drugs to counteract cartilage degradation during the most active phase. This chronobiological strategy maximizes therapeutic benefits, potentially slowing or halting early cartilage damage by directly addressing the circadian rhythm of metabolic activity. In contrast, Figure 3b depicts a “different-phase” approach, emphasizing drug administration during periods of minimal catabolic activity, such as during nighttime. Hypothetically, this misalignment results in a temporal mismatch between the peak bioavailability of the drug and the critical period of cartilage vulnerability, thereby compromising its protective efficacy. Misaligned delivery of chondroprotective agents, including glucosamine and chondroitin sulfate [86], may limit their ability to mitigate ECM breakdown effectively. By aligning drug administration with the morning degradation peak [20], we can enhance the efficacy of DMOADs, improving the management of early-stage OA and advancing therapeutic outcomes.

In light of the emerging role of circadian rhythms in joint health, particularly in relation to cartilage metabolism [20], it becomes increasingly clear that targeting these biological clocks holds significant potential for OA prevention and treatment. Specifically, the inter-relationship between circadian timing and chondroprotective therapy emphasizes its potential as a transformative strategy to optimize early OA interventions. Synchronizing drug administration with the natural diurnal/circadian biorhythms of cartilage offers a promising pathway to enhancing treatment efficacy and protecting cartilage health. However, achieving comprehensive and sustainable outcomes requires acknowledging that circadian timing alone cannot address the multifaceted nature of OA progression. Lifestyle factors such as PALs may play a similarly critical role in determining the effectiveness of therapeutic treatments [17,90]. The following sections examine these dimensions in greater detail, illustrating how an integrated approach that harmonizes circadian chronomedicine with physical and biochemical factors can drive more effective and holistic cartilage protection strategies.

### 6.4. Optimizing Drug Delivery in Cartilage: The Role of Synovial Fluid Dynamics and Joint Motion

The pharmacokinetic profile of chondroprotective agents—including their absorption, distribution, metabolism, and excretion (ADME)—is fundamental for achieving therapeutic concentrations in joint cartilage, directly influencing their effectiveness in tissue protection [163]. One critical trend emerging from the recent literature is the rapid clearance of DMOADs within the joint cavity [164], a key factor determining their therapeutic efficacy. The clearance of these compounds is a significant challenge that is largely dependent on their molecular weight, formulation, and pharmacological characteristics. Low-molecular-weight compounds (LMWCs), including low-molecular-weight drugs (LMWDs), typically exhibit a short residence time in the joint cavity, which limits their sustained therapeutic effects. For instance, small molecules such as glucosamine sulfate demonstrate a relatively rapid clearance, typically occurring within hours in both plasma and synovial fluid [95,97,100,165,166,167,168,169,170]. In contrast, larger biologics and viscosupplements such as hyaluronic acid may remain in the joint cavity for several days to weeks. This variability in clearance times is closely linked to the turnover rate of synovial fluid, which averages approximately one hour [100], complicating the maintenance of therapeutic drug levels within the synovial cavity.

The rapid elimination of most DMOADs is mainly driven by their low molecular weight, which enables efficient transport and removal from the diarthrodial joint to the systemic circulation, thus reducing their local therapeutic action in the cartilage [105,164,171]. This phenomenon is likely attributable to drug escape facilitated by the presence of a dense network of fenestrated lymphatic vessels situated close to the synovial membrane surface, which are oriented preferentially toward the joint cavity [100,172]. Upon drug delivery into the synovial cavity, small- and medium-sized anti-OA agents typically “leak” into the systemic circulation and fail to reach chondrocytes at therapeutic concentrations. This may partly explain the lack of effectiveness of traditional chondroprotective agents, including glucosamine and chondroitin sulfate, designed for articular cartilage, as they may be rapidly eliminated into the systemic circulation within hours and exhibit a limited penetration capacity in the chondral region [164]. Understanding these clearance dynamics is essential for optimizing dosing strategies and enhancing the clinical effectiveness of low-molecular-weight DMOADs in OA management, as maintaining adequate drug concentrations over time can significantly influence treatment outcomes.

In addition, orally administered DMOADs face significant challenges in penetrating the dense avascular structure of articular cartilage, thereby limiting their therapeutic efficacy in OA treatment [103]. The cartilage ECM, rich in collagen fibers and proteoglycans, acts as both a structural and biochemical barrier that significantly restricts the transport of therapeutic agents, particularly those with high molecular weights [92,101]. Adding to this complexity, the “unstirred layers hypothesis” highlights an often-overlooked pharmacokinetic challenge: a stagnant liquid film on the cartilage surface that acts as a diffusion barrier [17]. Although the notion of an unstirred layer adhering to the cartilage interface was first proposed by Maroudas et al. in the 1960s [30], recent studies have demonstrated that this film, characterized by immobilized solutes and a lack of fluid motion, further impedes the penetration of nutrients and drugs into the deeper cartilage layers [93]. This phenomenon limits passive solute diffusion into deeper cartilage zones, where chondrocytes depend on nutrient and drug delivery [17,90]. However, the thickness and concentration of this unstirred layer depend on the rate of fluid movement [30], particularly in the presence of biological viscous fluids, such as synovial liquid, in diarthrodial joints. These findings underscore the significance of physical activity in facilitating joint movement, which helps to disrupt stagnant layers and enhance solute diffusion, thus emphasizing the role of daily physical activity in promoting substance exchange. Aligning physical activity or exercise interventions with the pharmacokinetics of DMOADs—for instance, during peak plasma concentrations—may synergistically improve therapeutic outcomes by promoting drug diffusion and stimulating chondroprotective pathways [17]. Joint movement not only enhances synovial fluid mixing but also facilitates the transport of therapeutic agents into cartilage. Early studies demonstrated that small molecular compounds are primarily transported to chondrocytes through synovial fluid motion [30], whereas cyclic loading, such as walking, has been shown to enhance the movement of high-molecular-weight compounds through the cartilage matrix [92]. This evidence suggests that low-impact activities, such as pedaling, can enhance the absorption of smaller-molecular-weight DMOADs. Despite these promising findings, the optimal exercise parameters—frequency, intensity, time, and type (FITT)—for maximizing the therapeutic potential of DMOADs remain unclear. This highlights the urgent need for further research to establish evidence-based guidelines that integrate physical activity with pharmacological interventions, ultimately addressing drug delivery challenges, preserving cartilage integrity, and mitigating OA progression.

Considering the synovium as the “placenta” for avascular cartilage, leveraging synovial liquid as a vehicle for targeted pharmacological intervention in OA offers a promising and rational therapeutic strategy. Understanding the mechanisms underlying solute transport across cartilage and the role of joint movement has significantly advanced our knowledge of how nutrients, waste, and therapeutic agents are exchanged within the synovial joint [30,92,93,98,105,171]. However, the influence of synovial fluid dynamics on solute transport into cartilage has received limited attention in the literature [105]. This gap highlights the need to reconsider foundational concepts, particularly the mechanisms by which chondroprotective drugs penetrate cartilage and the role of synovial fluid motility in tissue retention [17]. Evidence indicates that drug penetration into cartilage is substantially reduced in immobilized joints, underscoring the critical role of joint movement in facilitating the diffusion of DMOADs. Specifically, joint motion reduces the impact of the stagnant liquid film at the cartilage–fluid interface [30,92,93], thereby enhancing drug diffusion and optimizing therapeutic outcomes. This growing understanding highlights the importance of synovial fluid dynamics in optimizing therapeutic outcomes and suggests that targeting these mechanisms may revolutionize OA management.

**Figure 4:**
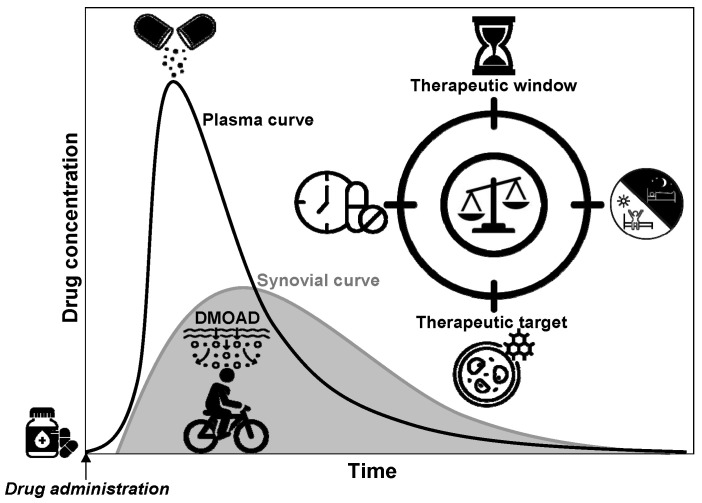
A scheduled approach for optimizing OA management by integrating physical activity with DMOADs at peak plasma drug concentrations. This figure illustrates the plasma (black line) and synovial (grey line) concentration–time profiles of orally administered LMWDs, such as glucosamine sulfate, highlighting the nonlinear pharmacokinetic elimination patterns. To optimize therapeutic effectiveness, bioavailability and pharmacokinetic data can be leveraged to target drug concentrations within the pharmaceutical window facilitated by joint movement. The figure shows the range of maximum plasma concentration (C_max_) values within the first 3 h, with a corresponding delay in peak synovial C_max_. This period represents the therapeutic window for overall drug exposure before the onset of the glucosamine sulfate elimination phase. Glucosamine is rapidly cleared (at a rate of 50 µL/min) through the lymphatic microvessels in the synovium [99,165,166]. However, drug diffusion into cartilage can be enhanced 2–4 h post-dose (1500 mg), as shown in the figure, by performing exercise such as pedaling for 30 min following glucosamine sulfate administration. Mechanical joint movement during cycling exercise ensures the continuous agitation of synovial fluid, creating a well-stirred condition that minimizes stagnant fluid films and facilitates drug diffusion across articular cartilage. Studies from [168,169] provide supporting data on the oral bioavailability of glucosamine. Although not shown in the figure, N-acetylcysteine, an acetyl derivative of the amino acid l-cysteine, demonstrates a similar pharmacokinetic profile, with the peak plasma concentration occurring approximately 2 h after oral administration [177].

From a clinical perspective, engaging in low-impact physical activities such as cycling may effectively promote synovial fluid mixing and enhance drug delivery to the cartilage [173,174]. These activities promote homogenization of the synovial cavity, reducing the influence of the unstirred layer and enabling deeper penetration of chondroprotective drugs into the cartilage [17]. Such improvements may lead to the accumulation of therapeutically significant drug concentrations in key joints such as the knee and hip. However, timing may play a critical role. The rapid clearance of LMWCs from the synovial cavity following oral administration necessitates initiating joint motion such as pedaling within the first three hours after drug intake to optimize synovial fluid mixing and drug retention, as shown in Figure 4. Nonetheless, it remains uncertain whether synovial liquid drug concentrations peak within this timeframe, as the equilibrium between plasma and synovial fluid may occur later. Studies in animal models, particularly those investigating glucosamine hydrochloride, have demonstrated a consistent temporal pattern of peak plasma and synovial glucosamine concentrations following oral administration [175]. However, further research is needed to fully understand the pharmacokinetics and clinical implications of these findings in humans, optimize dosing protocols, and assess inter-individual differences in absorption and therapeutic efficacy under diverse physiological and pathological conditions [176].

Although low-intensity pedaling at higher cadences shows promise for enhancing synovial mixing, additional studies are also required to determine the optimal types, intensities, and durations of joint movements, such as those involving the knee and hip, to maximize drug delivery. These findings highlight both a significant challenge in advancing innovative OA treatments and a valuable opportunity to refine and optimize traditional therapeutic strategies. Understanding how joint motion and exercise dynamically influence synovial fluid flow and how these changes mitigate the effects of unstirred layers on cartilage surfaces could help refine drug delivery strategies and improve DMOAD bioavailability. Addressing these physical barriers and integrating knowledge of the rheological properties of synovial fluid with pharmacological advancements may enable the development of novel approaches to delay the onset and progression of OA. Future clinical trials are essential to elucidate how variations in exercise parameters affect cartilage metabolism and drug penetration, with the goal of developing targeted and personalized physical activity/exercise regimens that incorporate timely and scheduled interventions for maximizing therapeutic outcomes.

### 6.5. CADENCE: Chondroprotection Advanced Through Deliberate Exercise and Networked Circadian Engagement

In the rapidly evolving field of OA management, a promising frontier emerges through the integration of chronobiology, chronoexercise, and chronopharmacology to optimize composition of synovial fluid. This holistic strategy has the potential to transform joint health by aligning therapeutic interventions with the natural circadian rhythm of the hyaline cartilage. Synovial liquid plays an essential role in maintaining cartilage integrity by acting as both a lubricant and carrier of vital nutrients and chondroprotective agents, while also aiding in the removal of pro-catabolic factors such as ECM fragments. During the early stages of OA, when cartilage metabolism remains adaptable, strategically timed interventions targeting the synovial fluid may help restore homeostasis and prevent disease progression. This perspective highlights the potential to synchronize physical activity/exercise regimens, pharmacological treatments, and nutritional therapies with the circadian rhythm of cartilage, thereby reducing low-grade inflammation, enhancing ECM repair, and slowing OA progression. Such an approach offers a promising new paradigm in cartilage health management within the expanding field of chronomedicine.

Cartilage exhibits a circadian rhythm that aligns with daily cycles of mechanical stress and repair: during the day, it endures high mechanical loads and increased metabolic demands, whereas nighttime provides a crucial period of reduced stress and enhanced reparative processes [130]. This cyclical pattern is essential for maintaining cartilage homeostasis and underscores the significance of coordinating therapeutic strategies with natural rhythms. The disruption of circadian clocks by environmental factors may contribute to cartilage degradation. Emerging evidence suggests that the circadian rhythm of cartilage regulates catabolic activity [20], indicating that key degradative processes, such as the breakdown of damaged proteins and ECM components, are synchronized to optimize cartilage homeostasis. Circadian regulation of cartilage metabolism ensures that catabolic processes, including proteasomal degradation and ECM turnover, peak in the morning [20]. This rhythmic synchronization allows chondrocytes to efficiently clear damaged proteins and maintain tissue integrity, prepare cartilage for the mechanical demands of daily activity, and mitigate the risk of premature degeneration. Interestingly, these findings suggest that rhythmic regulation of chondrocyte physiological processes may optimize cartilage metabolism, repair, and remodeling by aligning these activities at specific times of the day, potentially enhancing tissue maintenance and functional integrity.

**Figure 5:**
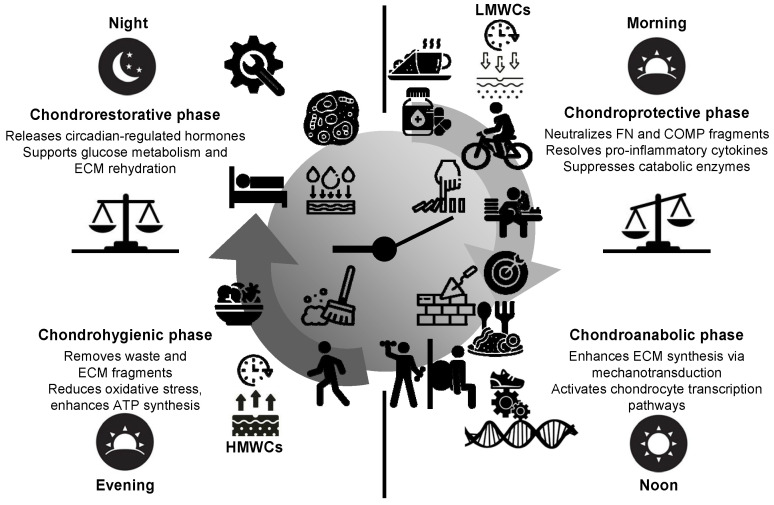
A schematic representation of the CADENCE model, which synergistically integrates circadian rhythms, targeted pharmacological interventions, and tailored exercise regimens into four interdependent phases to optimize cartilage preservation and delay OA progression. During the morning chondroprotective phase, agents such as glucosamine sulfate protect cartilage from pro-catabolic factors while supplying essential anabolic precursors. The noon chondroanabolic phase leverages exercise-induced mechanotransduction to stimulate ECM synthesis and promote anabolic activity. During the evening chondrohygienic phase, moderate physical exercise (e.g., walking) induces repetitive mechanical loading that reduces oxidative stress, promotes the clearance of matrix degradation products, and supports mitochondrial function. In the nighttime chondrorestorative phase, hormonal cycles and rest facilitate ECM rehydration, tissue repair, and the metabolic rebalancing of chondrocytes. These phases are hypothesized to be mutually reinforcing; for example, chondroprotective agents may not only shield cartilage from mechanical and biochemical stress but also provide critical building blocks for repair. Meanwhile, the “pumping effect” during the chondrohygienic phase may facilitate the mobilization of newly synthesized matrix components for proper deposition during nocturnal restoration, while early exercise-induced modulation of melatonin rhythms may further optimize the overall repair process. This interdependent synchronization of circadian, mechanical, and pharmacological inputs underscores the potential of the holistic approach of the CADENCE model to promote joint health and mitigate OA progression. Abbreviations: LMWCs, low-molecular-weight compounds; HMWCs, high-molecular-weight compounds.

These recent advances in the understanding of circadian regulation of cartilage physiology open new avenues for developing and optimizing therapeutic strategies to prevent early cartilage degeneration associated with OA. Increasing evidence supports the notion that aligning interventions—such as circadian-optimized DMOADs, antioxidant therapies, and time-specific physical activity/exercise (chronoexercise)—with the intrinsic biological rhythms of cartilage could enhance therapeutic outcomes and delay OA progression [17,18,19,20]. Despite this promising insight, the integration of circadian principles into a cohesive clinical framework remains underexplored. To bridge this gap, this study introduces the CADENCE model (Chondroprotection Advanced through Deliberate Exercise and Networked Circadian Engagement), which provides a structured framework for synchronizing pharmacological and non-pharmacological interventions with the circadian dynamics of joint cartilage. Table 2 presents a comprehensive overview of the CADENCE model-based framework that integrates therapeutic strategies, physical activity/exercise protocols, and timing recommendations to optimize cartilage preservation and support OA management. As depicted in Figure 5, this conceptual model outlines chrono-tailored phases—morning, noon, evening, and night—designed to leverage the optimal therapeutic windows to protect cartilage integrity. The CADENCE model offers a holistic approach to joint health by reducing catabolic activity, enhancing ECM synthesis, mitigating oxidative stress from ROS, and fostering cartilage repair. Although the segmentation of therapeutic phases is conceptual and may not fully represent the simultaneous nature of these processes, this model underscores the importance of circadian alignment as a strategic tool for optimizing cartilage-targeted therapies and for improving clinical outcomes in OA management.

As outlined previously, the CADENCE model synthesizes current evidence on circadian rhythms, physical activity/exercise, and the pharmacokinetics of DMOADs to present a novel approach for managing OA. The model delineates the daily physiological cycle into four phases: morning (chondroprotective phase), noon (chondroanabolic phase), evening (chondrohygienic phase), and night (chondrorestorative phase). Each phase is characterized by specific biochemical and biomechanical processes that regulate cartilage metabolism, thereby offering opportunities for targeted, time-sensitive interventions to optimize joint health. During the morning chondroprotective phase, when cartilage exhibits peak catabolic activity, the focus is on mitigating early catabolic events that trigger cartilage matrix breakdown. Dudek et al. [20] reported increased proteasomal degradation during the early hours, underscoring a critical period of vulnerability. Consequently, administering chondroprotective agents such as crystalline glucosamine sulfate [176], in combination with pharmaceutical-grade low-molecular-weight chondroitin sulfate and antioxidant supplementation—specifically N-acetylcysteine, a WHO-recognized antioxidant [197]—during this critical adsorption/absorption phase, may optimize their effectiveness in counteracting cartilage degradation.

The CADENCE model optimizes the chondroprotective effects of DMOADs by timing their oral administration to align with periods of peak cartilage sensitivity to mechanical stimuli [20,196]. Insights from Chen et al. [196] revealed that the three peaks in the mean step count on weekdays occur during specific time periods: one in the morning (8–10 a.m.), another over lunch (12–2 p.m.), and a third in the late evening (6–8 p.m.) for participants aged 30–59 years. Cyclic mechanical loading, such as that induced by walking, contributes to joint health by supporting tissue maintenance, including that of the capsuloligamentous complex and osteochondral unit. However, this mechanical loading may also accelerate the convective transport of large pro-catabolic molecules, such as fibronectin fragments and inflammatory cytokines, into deeper cartilage zones, potentially exacerbating matrix degradation [92,103,104,106,198]. As discussed earlier, the transport mechanism in cartilage depends on the size of the solute: smaller molecules diffuse passively, whereas larger compounds, such as high-molecular-weight cytokines, rely on convective fluid flow. Modeling studies of the intervertebral disc suggest that cytokines, such as TNF-α (MW: 17.5 kDa) and IL-1β (MW: 17.3 kDa), experience significantly enhanced transport under cyclic loading due to a “pumping effect”, which drives these macromolecules from the cartilage surface into deeper layers [199]. Timely inhibition of the activity of these cytokines is essential for preventing the activation of pro-catalytic enzymes that contribute to cartilage degradation.

In addition, antioxidant supplementation is integrated into the morning chondroprotective phase to support mitochondrial function in the deeper layers of cartilage, an essential aspect of maintaining redox balance and metabolic homeostasis within the joint tissue. Antioxidants, particularly N-acetylcysteine, have been shown to effectively counteract the detrimental effects of ROS by significantly reducing FN-fs-induced cartilage chondrolysis [69,70,143,200,201]. Homandberg et al. [69] demonstrated that FN-fs promote cartilage damage by enhancing MMP activity, suppressing proteoglycan synthesis, and generating ROS. Catabolic cytokines, such as TNF-α and IL-1, are central to this process. N-acetylcysteine effectively inhibited FN-fs-mediated proteoglycan depletion, reduced MMP-3 release, and promoted reparative responses, highlighting the potential of ROS-targeted therapies for mitigating cartilage degeneration. Further studies reported that N-acetylcysteine and glutathione facilitate the restoration of proteoglycan content in cartilage exposed to FN-fs [70]. These antioxidants neutralize ROS, suppress cytokine activity, and enable proteoglycan levels to recover to normal concentrations. Interestingly, the restoration of proteoglycans was dependent on the continuous presence of antioxidants, as their removal resulted in the re-emergence of proteoglycan depletion, suggesting that antioxidants primarily act to suppress cytokine activity rather than completely block their expression. These findings underscore the potential of antioxidants, particularly N-acetylcysteine, as therapeutic agents to combat cytokine-induced cartilage damage and to promote cartilage repair. The combination of glucosamine sulfate and N-acetylcysteine provides a promising strategy for reversing early cartilage damage mediated by FN-fs, ROS, and inflammatory cytokines, while also addressing mitochondrial dysfunction—a known contributor to ROS accumulation and cellular stress [143].

Antioxidant supplementation also plays a critical role in the CADENCE model, particularly in its potential to mitigate mitochondrial dysfunction and reduce ROS production in chondrocytes. Numerous studies have demonstrated that maintaining mitochondrial health is essential for preventing cartilage degeneration, as mitochondrial dysfunction is closely linked to elevated ROS levels, which can lead to chondrocyte apoptosis and accelerate cartilage breakdown [137,139,143,144,202,203,204,205,206]. The CADENCE model aims to optimize antioxidant delivery at times of increased oxidative stress to support mitochondrial resilience. Despite these promising findings, the efficacy of antioxidants in preventing cartilage degeneration remains controversial. Several studies report significant improvements in mitochondrial function and a reduction in oxidative damage following antioxidant supplementation [143], while others suggest that antioxidants alone may not sufficiently reduce ROS levels or fully prevent cartilage degradation [200]. This discrepancy highlights the need for a multifaceted therapeutic approach that combines antioxidants with additional interventions, such as physical activity/exercise or chondroprotective agents, to provide comprehensive protection and repair of the cartilage. Regular physical exercise has been shown to promote joint health by optimizing the homeostatic functions regulated by circadian rhythms within cartilage tissue [132]. Such consistent physical activity helps synchronize metabolic processes, improving mitochondrial function, reducing oxidative stress, and ultimately supporting cartilage integrity and preventing degeneration.

**Figure 6:**
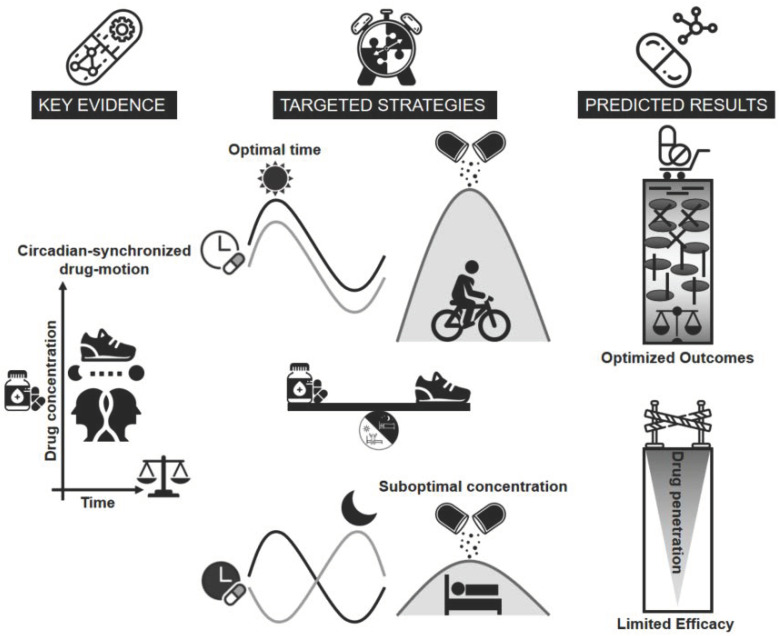
Two hypothetical scenarios illustrating the influence of circadian synchronization on drug delivery and joint activity. Top: During the morning chondroprotective phase, circadian-synchronized drug movement is optimized to address early catabolic events that contribute to cartilage matrix degradation. This is enhanced by strategically timed, coordinated joint activity—such as the sliding motion provided by cycling—that enhances drug penetration into the cartilage, particularly the deeper layers, thereby maximizing therapeutic efficacy and preserving joint health. Bottom: In the absence of circadian synchronization of drug movement, such as with oral administration during nighttime, the drug is less effective, resulting in poor diffusion across the cartilage. As a result, drug concentrations remain low, particularly in deeper tissue layers, limiting the effectiveness of the treatment and its potential to prevent further cartilage degradation.

As shown in Figure 6, the chondroprotective phase in the CADENCE model suggests that the pharmacokinetics of DMOADs follow an inverted Gaussian distribution [168,169], with peak plasma concentrations typically occurring in the morning. This time window coincides with increased joint motion, which is commonly induced by physical exercise. The synchronization of DMOAD administration with chronoexercise could enhance the intra-cartilage absorption of these agents, potentially inhibiting key catabolic cytokines such as TNF-α and IL-1, which are crucial mediators of matrix degradation [158,159,160,161,162]. However, the effectiveness of combining small DMOADs (e.g., glucosamine sulfate, MW: 179 Da) with bioactive dietary supplements (e.g., N-acetylcysteine, MW: 163 Da) or other diet-derived bioactive compounds [189,207,208], as summarized in Table 2, depends on their ability to reach the synovial fluid from the plasma and subsequently penetrate the avascular cartilage. The challenges of delivering drugs to the avascular cartilage—primarily related to the physical processes that precede the biochemical actions of drugs—are often overlooked in current therapeutic strategies. As previously mentioned, LMWCs rely on diffusion for transport, which is significantly enhanced by joint motion. Emerging evidence suggests that sliding motions may further accelerate the transport of small solutes [102,107]. Based on this, I hypothesized that cycling may improve the transport efficiency of chondroprotective agents into cartilage. Investigating the effects of knee and hip flexion–extension movements on drug transport could help optimize the therapeutic potential of these agents. Given the short residence time of LMWCs in the synovial cavity following oral administration [99,165,166], it is recommended to engage in pedaling within the first three hours after ingestion to promote effective mixing of the synovial fluid and facilitate sliding contact, thereby improving the penetration and retention of drugs within the dense cartilage matrix. This approach aims to maximize the efficacy of cartilage-preserving drugs in OA management, particularly in weight-bearing joints. While pedaling at a low-to-moderate aerobic intensity and higher cycling cadence can effectively stir synovial fluid and promote sliding contact, further research is necessary to determine the optimal joint motion patterns and dosing regimens to optimize therapeutic outcomes. This remains a critical area for future investigations into the prevention and treatment of OA.

Cycling may not only facilitate the diffusion of small protective molecules—such as glucosamine sulfate or N-acetylcysteine—into the cartilage matrix [17], thereby enhancing its structural and functional integrity, but could also trigger a range of therapeutic effects by stimulating key biological processes that might help preserve cartilage and optimize joint function. The cyclical flexion–extension movements inherent to cycling increase blood flow to articular tissues, induce capsular strain responses, and promote trans-synovial transport, collectively supporting the metabolic activity and health of synovial cells [95,96,146,209]. These mechanisms are particularly pronounced in type B synoviocytes, which are responsible for the synthesis of hyaluronic acid—a critical component of synovial fluid that ensures proper lubrication and viscosity. In fact, cyclic joint motion has been shown to increase hyaluronic acid production by 110–130%, thereby improving nutrient delivery to cartilage and reinforcing its biomechanical function [210,211,212,213]. Additionally, Moore and Burris [214] demonstrated that tribological rehydration—where sliding-induced hydrodynamic effects restore interstitial fluid—plays a key role in maintaining joint lubrication and preserving the cartilage structure. By promoting hyaluronic acid synthesis to mitigate morning joint stiffness, enhancing solute transport, and countering the effects of pro-inflammatory mediators, pedaling contributes to synovial fluid homeostasis, reduces low-grade inflammation, and protects cartilage from repetitive stress damage. Taken together, these findings underscore the potential of cycling as a low-impact exercise intervention during the chondroprotective phase of the CADENCE model—particularly in the early morning hours—to preserve cartilage health and slow OA progression.

Upon entering the chondroanabolic (afternoon) phase of the CADENCE model, cartilage exhibits a significant increase in ECM synthesis and tissue repair [132], primarily driven by the mechanotransduction machinery of chondrocytes during peak anabolic activity. This process, rooted in cartilage mechanobiology, is especially critical given the inherently limited regenerative capacity of cartilage. As shown in Table 2, resistance training and functional exercises can optimize cartilage circadian rhythms by enhancing mechanotransduction, which in turn promotes ECM production and strengthens cartilage integrity. Mechanotransduction—the conversion of mechanical stimuli into biochemical signals—plays a critical role in regulating chondrocyte activity, particularly in response to mechanical loading [58,61,62,194,215]. Moderate levels of exercise have been shown to influence the metabolomic profiles of chondrocytes, triggering anabolic pathways that support matrix synthesis and maintain tissue homeostasis [193,194,215]. This concept aligns with the principles of “mechanotherapy”, as described by Khan and Scott [183], which emphasizes how physical therapists’ exercise prescriptions promote tissue healing by utilizing mechanotransduction to drive tissue repair and remodeling in various musculoskeletal tissues, including cartilage. Functional exercises, such as squats, lunges, step-ups, and leg presses, combined with resistance training targeting weight-bearing joints, such as the knee and hip, provide controlled mechanical loading that activates anabolic processes in chondrocytes [194]. These exercises not only stimulate ECM synthesis to enhance cartilage repair but also improve joint stability and mobility [193]. Integrating these exercise regimens during the chondroanabolic phase of the CADENCE model can optimize cartilage repair, reduce degenerative changes, and help prevent OA progression by aligning therapeutic interventions with the natural circadian rhythm of cartilage, maximizing long-term joint health and functional outcomes.

Furthermore, mechanical stimuli derived from activities of daily living (ADLs) [196,216], particularly those occurring around noon, play a vital role in synchronizing chondrocyte circadian clocks and promoting ECM production, thereby supporting joint health. Circadian rhythms in cartilage are closely regulated by biomechanical forces, with rhythmic gene expression patterns driving anabolic processes essential for maintaining cartilage metabolism [125]. Even low-intensity mechanical loads, such as 0.5 MPa applied at 1 Hz, have been shown to synchronize chondrocyte circadian clocks, indicating that increased PALs, such as those from ADLs, provide a simple yet effective signal to support cartilage health [132]. These gentle weight-bearing activities induce repetitive mechanical loading, which synchronizes cartilage metabolism with the daily activity cycle, enhancing tissue repair and homeostasis while also preventing the detrimental effects of prolonged sedentary bouts, such as increased strain, reduced interstitial pressure, and higher friction on cartilage [217]. As illustrated in Figure 5, this effect is particularly evident in the afternoon, when tissue repair mechanisms are the most active [20]. Such temporal synchronization suggests that the circadian clock prepares chondrocytes for mechanical loading periods, aligning with the increased demand for protein synthesis. This adaptive mechanism enables cartilage to respond efficiently to mechanical stress and supports subsequent tissue repair [20,196]. Interestingly, many rhythmic cartilage proteins exhibit peak expression during the chondroanabolic phase, highlighting the potential adaptation of chondrocyte function to the 24 h day–night cycle.

The CADENCE model highlights the critical role of the chondrohygiene (evening) phase in preserving cartilage health and slowing OA progression by facilitating waste removal, reducing ROS, and promoting growth factor release for effective nighttime recovery with regular physical exercise, such as normal walking or gait [196], further optimizing cartilage rhythms, and enhancing ECM fragment clearance. As shown in Figure 5, this phase reduces oxidative stress and prevents mitochondrial dysfunction in chondrocytes. Accumulation of matrix degradation products, a hallmark of cartilage damage, is closely linked to the development and progression of OA. Mechanical stress, low-grade inflammation, and enzymatic activity contribute to the release of native COMP and FN from the ECM. These molecules are subsequently cleaved into smaller matrix degradation products that accumulate in the deeper cartilage layers, further disrupting tissue homeostasis and driving disease progression. Several studies on mechanical loading and cartilage metabolism have provided valuable insights into these complex processes. Mündermann et al. [191] observed fluctuations in serum COMP levels in healthy adults following a 30 min walking exercise. COMP levels increased by 9.7% 30 min post-exercise, returned to baseline within 3.5 h, and rose again by 7% at 5.5 h. Similarly, Andersson et al. [192] reported transient increases in serum COMP levels one hour after a one-hour treadmill walk, with levels normalizing within two hours. In addition, FN-fs, generated through mechanical stress and enzymatic cleavage by MMPs and ADAMTS, exacerbate cartilage breakdown by promoting the production of pro-inflammatory cytokines and matrix-degrading enzymes [218]. Among the FN-fs, the 29 kDa fragment is particularly destructive. This fragment exhibits potent catabolic activity, comparable to surgically induced cartilage damage, and freely diffuses through cartilage layers, accumulating in deeper regions [219]. These FN-fs clusters further impair cartilage integrity by disrupting joint lubrication mechanisms. The 29 kDa fragment binds to the lamina splenden, interfering with lubricating molecules such as lubricin, hyaluronic acid, and phospholipids. Likewise, COMP fragments have been shown to disrupt lubrication and exacerbate cartilage wear [220]. Collectively, these mechanisms underscore the detrimental effects of ECM degradation on cartilage homeostasis. These findings highlight the dual role of mechanical loading in cartilage metabolism, in which physiological loading supports matrix turnover and cartilage maintenance, but may also generate detrimental matrix degradation products that disrupt homeostasis, exacerbate cartilage degradation, and accelerate OA initiation and progression.

Although significantly elevated in the synovial fluid of OA patients, ECM fragments such as COMP and FN-fs are naturally present in vivo [179,191,221,222,223,224,225,226,227,228,229]. While these matrix degradation products are typically maintained in physiological balance, dysregulation can significantly increase the risk of OA by driving inflammatory processes. One prominent hypothesis suggests that COMP and FN fragments, as byproducts of cartilage degradation, play a key role in perpetuating inflammatory responses by activating the innate immune system [230]. Specifically, their accumulation in the synovial fluid triggers the release of pro-inflammatory cytokines, which exacerbates cartilage breakdown and contributes to joint degeneration. Furthermore, these ECM fragments are implicated in the activation of the complement pathway, a process that locally engages immune cells and perpetuates low-grade inflammation within the joint cartilage [231]. This establishes a pathological feedback loop, wherein subclinical inflammation accelerates further cartilage degradation, contributing to OA chronicity. Matrix degradation products, including COMP and FN fragments, also act as damage-associated molecular patterns (DAMPs), directly triggering inflammatory signaling pathways and exacerbating asymptomatic cartilage damage and silent joint inflammation [10,225,232]. Together, these mechanisms underscore the critical role of matrix degradation products in initiating and sustaining inflammatory cascades, perpetuating cartilage breakdown, and promoting OA progression.

**Figure 7:**
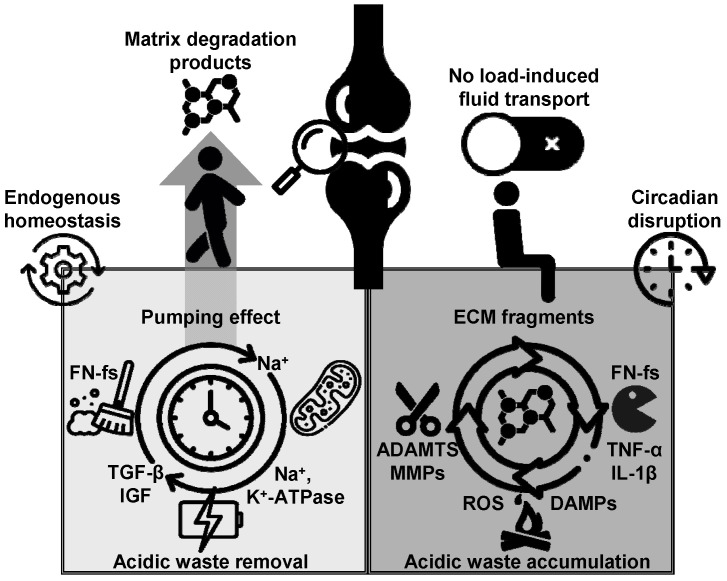
The effects of dynamic compression–decompression stimuli from mechanical loading, such as walking, on cartilage metabolism and homeostasis are shown in two hypothetical scenarios. Left: Dynamic compression induced by rhythmic mechanical loading stimulates ATP synthesis through biosynthetic pathways and enhances the convective transport of growth factors, enzymes, and hormones into the cartilage, primarily via Na^+^-dependent mechanisms. This process also facilitates the removal of matrix degradation products resulting from previous cartilage matrix fragmentation caused by weight-bearing activities. Overall, axial compression supports solute exchange, growth factor uptake, and ECM remodeling, promoting cartilage repair. Right: In the absence of mechanical loading, commonly associated with physical inactivity or a sedentary lifestyle, ECM fragments and acidic waste products, such as CO_2_ and lactate, accumulate due to the lack of desorption or exudation mechanisms provided by the pumping effect. This leads to oxidative stress, characterized by increased levels of reactive oxygen and nitrogen species, mitochondrial dysfunction in chondrocytes, and the release of matrix-degrading enzymes, all of which contribute to cartilage degradation and OA progression.

Figure 7 schematically illustrates that cyclic mechanical loading not only drives macromolecules deeper into the cartilage but also facilitates their clearance via fenestrated lymphatic vessels in the synovium, thereby removing ECM fragments and maintaining cartilage homeostasis. In contrast, sedentary behavior may impair these transport mechanisms, particularly in the deep zones of thick, weight-bearing cartilage in the knee and hip joints, leading to the accumulation of metabolic waste byproducts and pro-catabolic factors that exacerbate cartilage degradation [90]. Mechanical compression, such as that induced by evening walking [196], also generates fluctuations in free sodium concentrations and promotes water redistribution across regions of varying stiffness, thereby modulating ionic interactions within the pericellular matrix that are critical for effective mechanotransduction [53,56,58,60,61,62]. These Na^+^ fluctuations regulate the binding affinity and activation of growth factors—most notably TGF-β—in aggrecan-rich regions characterized by a high fixed charge density. In highly compressed areas, elevated sodium levels trigger the release and activation of growth factors that are indispensable for tissue maintenance and repair. Notably, cartilage contains a significantly higher sodium concentration than most tissues, underscoring its pivotal role in supporting load-dependent regulatory pathways that preserve tissue structure and function [57]. Osteoarthritic cartilage leads to a reduced fixed charge density and diminished Na^+^ levels, thereby disrupting these regulatory mechanisms and compromising cartilage integrity [60]. Furthermore, dynamic compression generates a “pumping effect” that enhances the convective transport of anabolic molecules—including growth factors, enzymes, hormones, and newly synthesized matrix macromolecules—into the cartilage [92,101], thereby delivering pro-anabolic factors such as collagen and proteoglycan precursors that are vital for ECM repair [233,234,235,236]. Collectively, these mechanobiological processes underscore the fundamental importance of mechanical loading and Na^+^-dependent regulatory pathways in preserving cartilage integrity by promoting tissue repair and facilitating catabolic debris clearance, thereby enhancing tissue cleaning and ultimately mitigating OA progression.

As the concluding stage of the proposed CADENCE model, the subsequent chondrorestorative phase represents a crucial window for supporting glucose metabolism in chondrocytes, the nocturnal secretion of anabolic and circadian clock-regulated hormones, and the natural rehydration of cartilage. As outlined in Table 2, these processes collectively restore ECM integrity, promote cartilage repair, and recover the biomechanical properties lost during daytime mechanical loading. Given that glycolysis is the primary ATP source for chondrocytes [136], circadian regulation of both transporters and glycolytic enzymes plays a critical role in cartilage metabolism. Interestingly, glucose metabolism peaks late at night, as highlighted by Dudek et al. [20] who demonstrated that several rate-limiting enzymes in the glycolysis pathway exhibit circadian rhythmicity. Notable examples include glucose-6-phosphate isomerase, which catalyzes the second step in glycolysis, and phosphoglycerate kinase, responsible for one of the two ATP-producing reactions in this pathway. Moreover, the activity of pyruvate kinase M, which oscillates between its highly active tetrameric form and nearly inactive dimeric form, governs whether glucose carbons are directed toward biosynthetic pathways or ATP production. It has also been observed that the rhythmic expression of the glucose transporter GLUT1 (Slc2a1) peaks in coordination with glycolytic enzyme activity [20]. Given that glycolysis is the primary source of ATP in chondrocytes [57,136], these circadian-regulated processes significantly affect cartilage metabolism and energy homeostasis. This rhythmic regulation is strongly linked to chondrogenesis, particularly during the nocturnal rest period, thereby emphasizing the importance of the chondrorestorative phase in maintaining cartilage health and promoting repair.

The nighttime period is characterized by the secretion of key anabolic and circadian-regulated hormones, including growth hormone, melatonin, thyroid-stimulating hormone, and cortisol [18,118,180,181,182]. Among these hormones, melatonin is a critical component of the antioxidative defense system, owing to its robust free radical-scavenging properties and its ability to enhance enzymatic antioxidant activity. Melatonin stimulates glutathione peroxidase, an enzyme essential for detoxifying hydrogen peroxide [237]. Notably, melatonin secretion is regulated by the circadian clock, with plasma levels peaking during late-night hours and falling to their lowest levels in the afternoon [238,239,240,241]. This rhythmic pattern highlights the key role of melatonin in promoting cartilage homeostasis during the nocturnal repair phase, as it helps mitigate ROS production, reduce the expression of pro-inflammatory cytokines, elevate the activity of matrix-degrading enzymes and support the maintenance of ECM integrity. These findings are consistent with the CADENCE model, which emphasizes the physiological role of circadian hormones, such as melatonin, in OA. Interestingly, further research has examined how physical exercise influences melatonin rhythms, revealing that both the timing and intensity of exercise can modulate these rhythms, potentially impacting cartilage repair processes. Miyazaki et al. [190] showed that a 2 h cycling session performed 3 and 7 h after waking advanced the onset of melatonin, helping the body adapt to the new schedule, while the sedentary group experienced a delayed phase of melatonin secretion. In contrast, Yamanaka et al. [195] found that cycling exercise for 2 h in the morning delayed the melatonin peak by one hour. These studies highlight the ability of exercise timing to modulate melatonin rhythms, which may, in turn, influence cartilage repair processes. Understanding how exercise affects melatonin secretion and cartilage homeostasis is essential for developing targeted therapeutic approaches such as circadian-stabilizing strategies—including sleep optimization and controlled light exposure—aimed at enhancing cartilage repair and slowing OA progression by aligning biological rhythms with restorative processes.

Moreover, melatonin plays a pivotal role in the regulation of the antioxidant defense system by stimulating key antioxidative enzymes. Sewerynek et al. [184] demonstrated a positive correlation between plasma melatonin levels and the total antioxidative capacity of the serum. This effect is mediated by the induction of enzymes, such as superoxide dismutase and glutathione peroxidase [184,237]. Glutathione peroxidase activity, in particular, exhibits a distinct circadian rhythm, with its peak occurring approximately 8 h after the onset of darkness and 4 h following the nocturnal rise in melatonin levels. This temporal relationship suggests that melatonin directly drives nocturnal upregulation of glutathione peroxidase activity. Similarly, Hasanoglu et al. [242] reported that the physiological nighttime surge in melatonin was closely associated with increased superoxide dismutase expression. Complementing melatonin, N-acetylcysteine enhances antioxidant defenses by acting as a precursor for glutathione synthesis, providing cysteine as the rate-limiting substrate and maintaining intracellular glutathione levels, which are critical for glutathione peroxidase activity [237]. Glutathione peroxidase detoxifies hydrogen peroxide, limiting oxidative damage, while glutathione reductase regenerates reduced glutathione, sustaining redox homeostasis. N-acetylcysteine has been shown to increase glutathione levels and upregulate glutathione peroxidase and superoxide dismutase activity, bolstering its capacity as a powerful scavenger of oxygen-derived free radicals [185,187,188]. Meanwhile, melatonin complements N-acetylcysteine by directly scavenging ROS and further enhancing the expression and activity of glutathione peroxidase, glutathione reductase, and superoxide dismutase. Taken together, these compounds have the potential to reduce oxidative stress, restore mitochondrial function, and modulate inflammatory responses. Additionally, N-acetylcysteine induces superoxide dismutase activity in immune cells and prevents oxidative damage-induced muscle dysfunction [186]. Hossain et al. [118] emphasized the dose- and time-dependent effects of melatonin in regulating ROS, oxidative stress, and anti-inflammatory responses, suggesting its potential to inhibit cartilage degradation by modulating pro-inflammatory cytokines. These studies emphasize the therapeutic potential of melatonin and N-acetylcysteine, individually or synergistically, in combating oxidative stress, preserving cartilage integrity, and mitigating OA progression. However, the dosing of melatonin remains controversial, with conflicting data suggesting both pro- and antioxidant effects depending on the dosage and context of its application.

The chondrorestorative phase also represents a critical period for cartilage rehydration, enabling the tissue to recover biomechanical properties lost during daytime compressive loading [21,98,243,244,245]. This rehydration process, facilitated by the metabolic equilibrium aligned with circadian hormonal activity, is driven by a proteoglycan-rich ECM, predominantly aggrecan, which exerts osmotic swelling pressure by attracting water through its densely packed, negatively charged glycosaminoglycans [49,50]. Sitoci et al. [51] examined nocturnal variations in knee cartilage thickness in healthy young adults using magnetic resonance imaging. Their findings showed a significant increase in cartilage thickness from evening to morning, with an average increase of 2.4% in the patella and 6.2–8.4% in the tibial regions. This suggests that knee cartilage recovers overnight, likely due to unloading during sleep. The study also observed that post-exercise cartilage deformation was generally greater in the morning than in the evening, although the difference was not statistically significant. These results indicate that cartilage thickness recovers by 2–8% overnight, regardless of sex [51]. The authors propose that the lack of pre-deformation in the morning, combined with potentially greater hydration after overnight unloading, may lead to a higher degree of post-exercise deformation in the morning than in the evening. This highlights the complex interplay among hydration, recovery, and mechanical loading in cartilage physiology. During loading, the cartilage expels water and deforms, redistributing the biomechanical stress across the joint surface [52]. Upon load release, the cartilage reabsorbs water and restores its original shape, demonstrating dynamic adaptation to mechanical demands. These diurnal fluctuations in water content—where cartilage loses water during daily mechanical loading and reabsorbs it during nocturnal rest—are vital for maintaining tissue hydration, supporting nutrient exchange, and preserving the mechanical functionality of the ECM. Collectively, these structural, biochemical, and electrochemical properties ensure cartilage resilience and functionality, enabling it to withstand repeated mechanical stress while preserving long-term joint health. By integrating these circadian-driven processes, the CADENCE model provides a comprehensive framework for promoting joint homeostasis and advancing therapeutic strategies aimed at preventing cartilage degeneration.

Unlike earlier methodologies, the CADENCE model introduces an innovative approach to OA management by integrating chronobiology with exercise and pharmacological therapies in a synchronized manner. Previous strategies, such as the BART (Beating osteoARThritis) program [246], emphasized a stepped-care model that prioritizes self-management through exercise and dietary changes before progressing to more intensive treatments, thereby highlighting the crucial role of lifestyle in managing OA. Both the CLIP-OA and POWER trials focused on combining exercise with weight management interventions to address knee OA in overweight or obese patients [247,248]. The CLIP-OA study protocol compared a personalized exercise and dietary weight loss program with the Arthritis Foundation’s Walk with Ease program, emphasizing mobility, weight loss, and cost-effectiveness [247]. Similarly, the POWER trial investigated the efficacy of a physiotherapist-delivered diet and exercise program versus exercise alone with the aim of assessing weight loss outcomes and improvements in knee pain and function [248]. Both randomized controlled trials highlighted the importance of integrated and accessible interventions to improve clinical outcomes. Further research supports the effectiveness of combining exercise and educational interventions to improve physical activity levels and reduce pain in patients with hip and knee OA. Okita et al. [249] conducted a systematic review and meta-analysis, demonstrating that combination therapy, which includes both exercise and educational components, leads to improvements in physical activity and modest pain reduction, although the overall evidence remains of low to very low quality owing to biases in study design. This supports the approach adopted in programs such as MultiKnee [250], which similarly integrates education and exercise to comprehensively address both the physical and psychological aspects of knee OA. The CLEAT (CycLing and EducATion) program [251], which specifically incorporates cycling as a form of low-impact exercise, has demonstrated effectiveness in managing hip OA, showcasing the potential benefits of cycling as an accessible and therapeutic modality. While these programs emphasize the importance of exercise and education, the CADENCE model uniquely incorporates circadian principles, providing a framework for optimizing treatment outcomes by synchronizing interventions with the natural circadian rhythms of cartilage. This alignment of exercise and pharmacological treatments with chronobiology provides a promising new direction for OA care, enabling a more personalized and potentially more effective approach. This approach is supported by chronotherapy strategies in the context of inflammatory joint diseases [252,253,254], where circadian biology has been shown to enhance the efficacy of treatments such as glucocorticoids (e.g., prednisone) and methotrexate by synchronizing their administration with inflammatory cytokine rhythms. Therefore, CADENCE may represent a significant step forward in optimizing OA management by integrating the strengths of established approaches with cutting-edge chronobiological insights.

In summary, emerging evidence underscores the pivotal role of circadian rhythms in cartilage biology, redefining our understanding of hyaline articular cartilage as an active temporally regulated tissue essential for joint homeostasis and function. The oscillatory activity of the chondrocyte circadian clock governs diurnal variations in anabolic and catabolic processes, with significant implications in preventing cartilage degeneration and managing OA. Building on these insights, innovative strategies such as chronoexercise—aligning physical activity/exercise with circadian peaks in cartilage responsiveness—have been proposed to enhance cartilage repair and delay OA progression by optimizing the biomechanical and biochemical environment of chondrons. Similarly, integrating chronotherapy into OA management, wherein chondroprotective drugs are administered in synchrony with circadian rhythms and joint motion, has the potential to boost therapeutic efficacy, reduce side effects, and support cartilage resilience. In this context, the CADENCE approach offers an innovative framework that synchronizes pharmacotherapy, exercise, and cartilage responsiveness to stabilize the ECM, mitigate oxidative stress, and address mitochondrial dysfunction, all key drivers of OA pathophysiology. By combining circadian-stabilizing strategies with precisely scheduled low-impact exercises such as cycling, the CADENCE model represents a transformative paradigm for joint health management, potentially delaying OA onset and preserving cartilage functionality while paving the way for personalized preventive chronomedicine.

## 7. Limitations of This Study

This study highlights several critical limitations in the current understanding of circadian rhythm regulation, chronoexercise, and chondroprotective agents for OA management. One of the most significant challenges is the lack of standardized methodologies for synchronizing therapeutic interventions, such as exercise timing and drug administration, with circadian rhythms, resulting in inconsistent outcomes and difficulties in cross-study comparisons. While the CADENCE model conceptually segments these interventions into distinct phases for illustrative purposes, it is essential to recognize that many of these processes naturally overlap or occur simultaneously throughout the day. Despite its promise, the clinical applicability of this chrono-tailored ‘prototype’ remains largely unexplored. Although CADENCE offers an innovative framework for synchronizing physical activity and pharmacotherapy with circadian biology, its practical application in the clinical setting has yet to be examined. Real-world challenges, including adherence to circadian-aligned regimens among individuals with varying chronotypes, PALs, lifestyle risk factors, comorbidities, and disrupted circadian patterns, require further investigation to establish their feasibility and efficacy. Furthermore, the pharmacokinetics and circadian dynamics of chondroprotective agents remain poorly understood, impeding efforts to maximize their therapeutic potential via time-sensitive delivery. Differences in the pharmacokinetic profiles of glucosamine and N-acetylcysteine, which exhibit a minimum one-hour difference in peak plasma concentrations [168,169,177], present a challenge in scheduling the timing of physical exercise to effectively mobilize the diarthrodial joint and maximize drug penetration into the cartilage. Defining the optimal FITT for physical exercise programs to maximize trans-synovial drug permeation, minimize diffusion barriers, improve drug penetration, and ensure sustained drug retention and uptake in the cartilage remains an underexplored area of research. Moreover, reliance on specific pharmacological agents such as glucosamine, chondroitin sulfate, and N-acetylcysteine also assumes their universal availability and accessibility, which may not be feasible in all healthcare systems or socioeconomic contexts. The controversy surrounding the clinical effectiveness of widely used chondroprotective agents such as glucosamine and chondroitin sulfate further complicates their integration within circadian-based approaches [6,7,9,14,15,16]. Inconsistent evidence, driven by variability in study designs, dosages, and patient demographics, raises questions about their role in optimizing OA management. Effectively managing OA requires more than merely cartilage care, as simplistic and reductionist strategies have proven insufficient [12]. It demands a comprehensive approach that addresses all potential risk factors, recognizing OA as a complex disease that affects not only the entire articular organ but also systemic physiology. Thus, a multifaceted approach is required that also accounts for additional osteoarticular risk factors, such as obesity, muscle weakness, joint laxity, injuries, and genetic predispositions [11]. Another notable limitation is the reliance on cross-sectional and preclinical studies, which fail to capture the cumulative long-term effects of circadian-aligned interventions on OA progression. The under-representation of diverse populations in interventional trials, along with the variability of circadian rhythms influenced by factors such as age, sex, genetics, and environmental conditions, further limits the generalizability of existing findings. Additionally, the interplay between circadian biology, exercise, nutrition, and pharmacology remains inadequately characterized, with a limited understanding of how these factors may synergize—or conflict—in therapeutic contexts. Despite considerable research efforts, reliable and specific biomarkers of OA remain elusive. Current combinations of chemical biomarkers and imaging techniques often lack specificity and reliability, making it difficult to assess cartilage health, monitor treatment efficacy, or detect the early progression of OA. The absence of robust biomarkers hampers not only the development of effective diagnostic tools but also the optimization of therapeutic interventions, such as DMOADs, for targeted and personalized care. It is also important to integrate these limitations into cartilage chronobiology studies, including investigations of clock regulation and the desynchronization (i.e., attenuation) of circadian rhythms in both the central clock and peripheral clocks. A significant limitation of this study is its omission of the influence of the circadian clock on other intra-articular tissues, such as the synovium, and extra-articular tissues, such as muscle, both of which are crucial for overall joint health and may considerably affect therapeutic outcomes, considering the multifaceted nature of OA management [12]. To address these challenges, future research should prioritize large-scale, longitudinal, and multicenter studies that include diverse populations and standardized protocols. Exploring the mechanistic links between circadian regulation, cartilage biology, and OA progression through innovative omics-based approaches, as well as by rigorously testing the CADENCE model in clinical settings, will be crucial. By refining chronoexercise protocols, advancing our understanding of circadian pharmacokinetics, and identifying reliable biomarkers, the field can move closer to personalized, evidence-based strategies that optimize OA management while improving patient outcomes.

## 8. Conclusions and Directions for Future Research

Despite these limitations, this study underscores the transformative potential of integrating circadian biology into comprehensive OA management by proposing the CADENCE model—a reimagined framework that synchronizes circadian rhythm regulation, physical activity/exercise programs, and chondroprotective agents—to improve cartilage health and therapeutic efficacy. Although promising, its clinical applicability remains unproven and requires rigorous validation using well-designed methodologies in real-world clinical settings. Future research should conduct longitudinal studies and randomized controlled trials (RCTs) with appropriate control groups (receiving standard OA treatments), double-blind procedures, and follow-up periods of 12 to 24 months to evaluate both short- and long-term impacts on cartilage health while controlling for potential confounders using advanced statistical models. Moreover, future research should focus on mapping circadian fluctuations in cartilage biology, such as variations in proteoglycan synthesis, the turnover of non-collagenous proteins, and inflammatory marker levels, to identify the optimal time windows for therapeutic interventions that enhance exercise protocols and drug delivery. Similarly, optimize exercise protocols by tailoring FITT parameters to target periods of peak cartilage responsiveness. The development of specific, validated biomarkers, coupled with advanced imaging techniques, such as ultrastructural cartilage imaging, will further enable the precise monitoring of treatment-induced changes in cartilage health and disease progression. Addressing one of the greatest challenges in OA management, future research should also focus on overcoming the limitations of delivering drugs to the avascular cartilage. Innovations in nanoparticle-based delivery systems hold promise for optimizing the chronopharmacokinetics of chondroprotective agents by ensuring sustained release and enhanced retention, as well as by extending the half-lives of LMWD—which typically exhibit short half-lives—and may facilitate the co-delivery of multiple drugs in fixed-dose combinations to improve adherence and therapeutic outcomes, although these systems require validation through comprehensive preclinical studies, multicenter clinical trials, and RCTs [255]. Furthermore, the integration of precision medicine—tailoring treatments based on individual genetic profiles, comorbidities, and lifestyle factors—holds the potential to personalize OA therapies and identify patient subgroups most likely to benefit from circadian-based approaches. In sum, as we push the boundaries of personalized chronomedicine, it is increasingly evident that chronotherapeutic strategies may become a cornerstone of cartilage care [129], bringing us closer to a future in which therapeutic interventions are precisely tailored to maximize cartilage protection and joint health, offering hope for more effective and sustainable OA management.

## Figures and Tables

**Table 2:** Guidelines for optimizing cartilage health and OA management based on the CADENCE framework.

Phase	Therapeutic Strategy *	Exercise Protocol (FITT) **	Timing, Recommendations, and Rationale
Chondroprotective	Small DMOADs (e.g., glucosamine sulfate, LMW chondroitin sulfate, and small-molecule ADAMTS/MMP inhibitors) and bio-active dietary supplementation (e.g., N-acetylcysteine, omega-3 fatty acids, small collagen-like peptides, resveratrol, polyphenols, sulforaphane, avocado/soybean unsaponifiables, and natural drugs/remedies with good safety profiles, such as Boswellia serrata, Devil’s claw (Harpagophytum procumbens), turmeric curcumin, and ginger), as well as the Mediterranean diet and its phytochemical components (e.g., flavonoids, polyphenols, and carotenoids) that have anti-inflammatory and antioxidant properties, may complement and support joint health. Functional foods, such as fortified breakfast items, dietary fiber, and probiotics, can be incorporated to deliver targeted chondroprotective benefits and further enhance cartilage integrity	Frequency: 6–7 days/week; Intensity: Low-to-moderate pedaling cadence (90–110 RPM) with minimal resistance (<40% VO_2_ max), performed either continuously or intermittently; Time: 30–45 min; Type: Cycling at high cadence	Morning (7:00–9:00 AM): 1–3 h post-intake, coinciding with peak drug pharmacokinetics and circadian-driven catabolic activity. This timing optimizes fluid mixing, promotes vital nutrient and small drug delivery, and enhances the sliding motion, all while minimizing joint stress
Chondroanabolic	Mechanotherapy to support ECM synthesis	Frequency: 3–4 days/week; Intensity: Moderate; Time: 30–40 min; Type: Resistance training (e.g., isotonic, isometric and isokinetic) and functional exercises	Midday (12:00–2:00 PM): Supports ECM synthesis via mechanotransduction during peak anabolic activity. Promotes cartilage repair and resilience by enhancing glycosaminoglycan, non-collagenous proteins, and other ECM components
Chondrohygienic	Focus on cyclic mechanical loading (e.g., walking) to facilitate waste removal and promote mitochondrial health	Frequency: 6–7 days/week; Intensity: Moderate cyclic loading (e.g., 2.8 MPa at 1 Hz) *; Time: 45–60 min; Type: Walking or low-impact cyclic loading exercises	Evening (6:00–8:00 PM): Supports ECM turnover and metabolic waste removal (e.g., lactate and CO_2_) via cyclic mechanical loading and convective transport. Regulates ion homeostasis via Na^+^/K^+^-ATPase activity and Na^+^-dependent mechanisms, sustaining chondrocyte function. Reduces oxidative stress, enhances mitochondrial efficiency, and primes cartilage for nighttime recovery. Mechanical loading, including axial compression, also activates TGF-β, triggering the release of ECM-sequestered growth factors critical for cartilage repair
Chondrorestorative	Prioritize rest and recovery to support rehydration and ECM restoration. Optional supplements (e.g., melatonin, tryptophan and magnesium) can aid in aligning circadian rhythms	Rest phase—no exercise	Night (9:00–11:00 PM): Hormonal regulation (e.g., melatonin, IGF-1) supports ECM restoration, glucose utilization and and proteoglycan synthesis. Sleep optimizes cartilage repair, aided by supplements taken with dinner for circadian alignment and metabolic efficiency

* Studies from ref [6,7,8,69,70,73,76,77,78,79,80,81,82,83,84,85,86,87,88,178,179,180,181,182,183,184,185,186,187,188,189] provide supporting data included in the table. ** The studies in ref [30,92,100,102,107,132,145,146,183,190,191,192,193,194,195,196] provided valuable data on FITT parameters, but standardized guidelines and tailored protocols for OA remain scarce. ref [168,169] and ref [177] further support the pharmacokinetics of glucosamine sulfate and N-acetylcysteine, aligning with the circadian timing recommendations presented in the table. Abbreviations: VO_2_ max: Maximum oxygen uptake during aerobic exercise, expressed in mL/kg/min, indicating exercise intensity.

## Data Availability

Not applicable.
