# Correction: del Río, E. Rethinking Osteoarthritis Management: Synergistic Effects of Chronoexercise, Circadian Rhythm, and Chondroprotective Agents. *Biomedicines* 2025, *13*, 598

**DOI:** 10.3390/biomedicines13092204

**Published:** 2025-09-09

**Authors:** Eloy del Río

**Affiliations:** Independent Researcher, 11520 Cádiz, Spain; eloy.delrio@uca.es

## References

The retracted reference 113 was deleted from the original paper. With this correction, the order of some references has been adjusted accordingly. The authors state that the scientific conclusions are unaffected. This correction was approved by the Academic Editor. The original publication [1] has also been updated.
